# Subphenotypes and the De Ritis ratio for mortality risk stratification in sepsis-associated acute liver injury: a retrospective cohort study

**DOI:** 10.1016/j.eclinm.2025.103173

**Published:** 2025-03-27

**Authors:** Lars Palmowski, Britta Westhus, Andrea Witowski, Hartmuth Nowak, Isabella Traut, Ali Canbay, Andreas Schnitzbauer, Paul Elbers, Michael Adamzik, Antonios Katsounas, Tim Rahmel

**Affiliations:** a Department of Anesthesiology, Intensive Care and Pain Therapy, University Hospital Knappschaftskrankenhaus Bochum, In der Schornau 23-25, Bochum 44892, Germany; b Department of Internal Medicine, University Hospital Knappschaftskrankenhaus Bochum, In der Schornau 23-25, Bochum 44892, Germany; c Department of Visceral, Oncological, and Transplant Surgery, University Hospital Knappschaftskrankenhaus Bochum, In der Schornau 23-25, Bochum 44892, Germany; d Department of Intensive Care Medicine, Center for Critical Care Computational Intelligence (C4I), Amsterdam Medical Data Science (AMDS), Amsterdam Cardiovascular Science (ACS), Amsterdam Institute for Infection and Immunity (AII), Amsterdam Public Health (APH), Amsterdam UMC, Vrije Universiteit, De Boelelaan 1117, Amsterdam 1081 HV, the Netherlands

**Keywords:** Acute liver injury, Sepsis, De Ritis ratio, SALI, Phenotype, Subphenotype

## Abstract

**Background:**

Sepsis-associated liver injury (SALI) is associated with poor outcomes and increased mortality. However, effectively stratifying SALI patients according to prognosis remains challenging. This study evaluates laboratory-based clustering filters for stratifying SALI patients by 30-day mortality risk, utilizing data mining techniques for novel pattern discovery.

**Methods:**

This retrospective cohort study analyzed SALI patients from two ICU databases: Medical Information Mart for Intensive Care (MIMIC)-IV database (n = 73,181, study period: 2008 to 2019) and Amsterdam UMC (n = 16,194, study period: 2003 to 2016). Patients were identified using Sepsis-3 criteria and liver injury markers. Risk stratification employed three laboratory-based approaches: (I) De Ritis ratio (aspartate aminotransferase/alanine aminotransferase), (II) R-factor (alanine aminotransferase and alkaline phosphatase relative to their upper limits of normal), and (III) alanine aminotransferase elevation. Kaplan–Meier analysis and multivariable Cox regression assessed the association between stratification methods and 30-day mortality risk.

**Findings:**

SALI patients had almost a 2-fold higher risk of 30-day mortality than those without SALI (hazard ratio: 1.73; 95%-CI: 1.58–1.90, p < 0.0001). Each stratification method (I-III) successfully classified patients into statistically distinct risk strata. The De Ritis ratio emerged as the strongest prognostic differentiation method: a ratio ≤1 indicated no significant increase in mortality risk (hazard ratio: 0.86; 95%-CI: 0.68–1.09, p = 0.21), whereas ratios of 1–2 and ≥2 were significantly associated with higher mortality (hazard ratio: 1.56; 95%-CI: 1.37–1.78, p < 0.0001 and hazard ratio: 2.46; 95%-CI: 2.18–2.77, p < 0.0001, respectively). All findings were confirmed in the validation cohort.

**Interpretation:**

The De Ritis ratio serves as a valuable prognostic tool for 30-day mortality in SALI patients. Our findings indicate that patients with a ratio ≥1 face significantly worse outcomes, highlighting the need for targeted interventions. These results refine risk stratification in SALI subphenotypes, enhancing our understanding of its prognostic implications.

**Funding:**

This study received no external funding and was solely financed through the departmental resources of the authors.

**Research in context**

**Evidence before this study**

We systematically searched PubMed and Web of Science up to November 2024 using the following search terms: “sepsis-associated liver injury” “SALI”, “De Ritis ratio”, “R-factor”, “ALT”, “mortality prediction”, “risk stratification”, and “sepsis”. We included studies that investigated liver injury in sepsis, its prognostic implications, and potential biomarkers for risk stratification. Only peer-reviewed articles in English and German were considered. Our search revealed that sepsis-associated liver injury (SALI) is a well-recognized complication of sepsis, associated with increased morbidity and mortality. However, current approaches for risk stratification remain inadequate. Yet, their application in SALI has not been validated, and no study has directly compared their predictive performance in this context. This gap underscores the need for improved subphenotyping and risk stratification in SALI.

**Added value of this study**

This study is the first to systematically evaluate and compare the De Ritis ratio, R-factor, and alanine aminotransferase for risk stratification in SALI using two large critical care databases (Medical Information Mart for Intensive Care (MIMIC)-IV database and Amsterdam UMC database). We identify the De Ritis ratio as the most robust predictor of 30-day mortality, categorizing patients into low (≤1), intermediate (>1 to <2), and high (≥2) risk groups. Our findings demonstrate that the De Ritis ratio reliably distinguishes a SALI subphenotype with no increased mortality risk, addressing a critical gap in existing research. Unlike other markers, the De Ritis ratio offers a simple, clinically accessible, and cost-effective tool for early risk stratification in critically ill patients with SALI.

**Implications of all the available evidence**

Our findings highlight the De Ritis ratio as a practical and effective tool for mortality risk stratification in SALI, with potential to enhance ICU patient management and therapeutic decision-making. Patients with a De Ritis ratio ≥2 may require intensive monitoring and early interventions, while those ≤1 can be managed with standard supportive care. Given the observational nature of our study, further prospective validation in diverse populations is essential to confirm its broader applicability. Future research should also explore the molecular and genomic underpinnings of SALI subphenotypes to refine precision medicine approaches in sepsis-related organ dysfunction.

## Introduction

Sepsis often leads to multi-organ failure,^1^^,^^2^ with the liver being among the severely affected organs, a condition known as sepsis-associated liver injury (SALI).^3^ SALI manifests in various clinical forms—such as jaundice, coagulopathy, and elevated liver enzymes –, all of which indicate functional deterioration and significant hepatocellular injury.^4^ The diverse aetiologies of SALI, including bacterial or viral infections, hypoxia, and drug-induced liver injury,^5^ along with its different forms of injury (cholestatic versus hepatocellular) variably impact prognosis in SALI patients, thereby complicating clinical management of sepsis.^3^^,^^6^^,^^7^

Despite the critical impact of liver dysfunction in sepsis, there is currently no standardized method for stratifying SALI patients based on their mortality risk using routine laboratory parameters.^4^ Paradoxically, while laboratory markers such as serum transaminases, bilirubin levels, and the international normalized ratio (INR) provide valuable insights into the different forms liver damage, their prognostic utility in SALI remains widely underexplored.^4^^,^^8^ Furthermore, biomarker ratios, including the De Ritis ratio, which compares aspartate aminotransferase (AST) to alanine aminotransferase (ALT) levels,^9^ and the R-factor, which assesses liver injury patterns by evaluating the ratio of ALT to alkaline phosphatase (ALP),^10^ along with the degree of ALT elevation in serum,^11^ are essential for assessing risk and classifying acute liver injury (ALI). However, their value in identifying subphenotypes in SALI patients remains controversial. Moreover, these assessments have not been validated for risk stratification in SALI nor adequately compared in the context of sepsis.^9^^,^^12^

This study aims to define clinical subphenotypes of SALI by applying laboratory-based risk stratification methods and assess their association with 30-day mortality. Here, we focus on adapting and evaluating established prognostic methods from non-septic acute liver failure, including the De Ritis ratio, R-factor, and elevated ALT levels. By applying these approaches to SALI, we aim to assess their prognostic value and enhance early identification of SALI subphenotypes at high risk for poor outcome.

## Methods

### Study design and conceptual overview

In this retrospective study, we analyzed two large and publicly available cohorts of intensive care unit (ICU) patients. The derivation cohort (DC) for our study was sourced from the Medical Information Mart for Intensive Care (MIMIC)-IV database. MIMIC-IV is a publicly available, de-identified dataset that contains detailed clinical information on 73,183 ICU admissions at the Beth Israel Deaconess Medical Center in Boston, Massachusetts, spanning the period from 2008 to 2019. This comprehensive resource includes patient demographics, vital signs, laboratory results, medications, procedures, diagnoses, and survival outcomes, thereby providing a robust platform for clinical research in critical care settings.^13^ The validation cohort (VC) was sourced from the Amsterdam UMC database, comprising 16,194 ICU cases spanning the study period from 2003 to 2016.^14^ Initially, 16,075 patients in the DC and 4881 patients in the VC were identified based on the Sepsis-3 criteria.^1^^,^^15^ Subsequently, patients without available laboratory values for total bilirubin, ALP, serum activity of ALT and AST within the first seven days were excluded. In the derivation cohort, patients with pre-existing mild, moderate or severe liver disease were also excluded.^16^ This approach resulted in the selection of 12,716 sepsis cases in the derivation cohort and 4538 sepsis cases in the validation cohort, all of which underwent screening for SALI as depicted in our study flow chart (Supplementary Fig. S1). The primary endpoint of our analysis was 30-day mortality.

### Ethics

This study utilized de-identified patient data from the MIMIC-IV and AmsterdamUMCdb databases. Ethical approval for MIMIC-IV was granted by the Institutional Review Boards of the Massachusetts Institute of Technology and Beth Israel Deaconess Medical Center. The AmsterdamUMCdb was developed in compliance with EU General Data Protection Regulations. Given these approvals and the exclusive use of anonymized data, no additional local ethical approval was required. Furthermore, as all patient data in both cohorts were fully anonymized prior to access, and due to their retrospective nature, the requirement for individual informed consent was waived. The use of these datasets complies with all relevant ethical guidelines and regulatory requirements.

### Screening for SALI

To account for the pathophysiologic alterations in sepsis, we applied Modified Drug-Induced Liver Injury (DILI) Criteria to identify patients with SALI within the first seven days after sepsis diagnosis (i.e., upon the first fulfilment of Sepsis-3 criteria).^1^^,^^17^ SALI was considered present if at least one of the following conditions was met: an ALT level of at least five times the upper limit of normal (ULN); an ALP level of at least twice the ULN; a combination of serum total bilirubin of at least 2.0 mg/dL and ALT at least three times the ULN; or a combination of serum total bilirubin of at least 2.0 mg/dL and ALP at or above the ULN.

### Stratification of SALI

To classify SALI severity, we established three distinct risk categories based on the De Ritis ratio, the R-factor, and serum ALT levels. The De Ritis ratio, defined as the ratio of serum AST to serum ALT levels, was used to categorize patients into three groups: ≤1, >1 and < 2, and ≥2. The R-factor, calculated as the ratio of ALT divided by its ULN to ALP divided by its ULN, was used to define the categories R < 2, R ≥ 2 and < 5, and R ≥ 5.^17^ Finally, stratification based on ALT levels categorized patients into those with ALT <2 ULN, ALT ≥2 and < 5 ULN, and ALT ≥5 ULN.^18^ This approach was formed by a comprehensive review of existing literature and validated clinical methodologies, ensuring that our approach was grounded in empirical evidence and expert consensus. In particular, our supervised risk classification approach, based on data mining techniques, identifies subphenotypes based on mortality clusters using established laboratory parameters, which can be arbitrarily modified in the context of SALI.

### Statistics

Continuous variables were summarized as means with standard deviations (SD) if they followed a normal distribution, or as medians with interquartile ranges (IQR; 25th to 75th percentile) for non-normal distributions. To assess the normality of continuous variables, we employed both visual (histogram and QQ-plot) and statistical (Shapiro–Wilk test) methods. Homogeneity of variance was assessed using Levene's test before applying independent t-tests. Differences between groups for continuous variables were analyzed using either the independent t-test or the Wilcoxon rank-sum test, depending on the distribution. Categorical variables were compared between groups using Fisher's exact test. Survival probabilities over time were estimated using Kaplan–Meier survival curves, with differences between these curves assessed statistically using the log-rank test. To evaluate the impact of various predictors on survival, multivariable Cox regression analysis was performed adjusted for demographics, comorbidities and disease severity at admission, as recommend for routine electronic clinical data.^19^ For survival analysis, we assessed the proportional hazards assumption using Schoenfeld residuals and checked the linearity of continuous predictors with Martingale residuals. When violations of the proportional hazards’ assumption were detected, specifically for SOFA score and age, we incorporated time-dependent effects into the Cox model to account for their variation over time. Confounders were selected based on clinical relevance, prior literature, and the disjunctive cause criterion, ensuring that all variables influencing either the exposure or the outcome were included while avoiding overadjustment. For the survival analysis, both the origin and start time were defined as the time of sepsis diagnosis, ensuring a consistent time zero across all patients. All individuals entered the study at this predefined time point, and no delayed entry occurred. Follow-up continued for 30 days after sepsis diagnosis. In this study, survival follow-up was formally available for all patients until day 30. Mortality status was determined either during the hospital stay or through linkage to the State Registry of Vital Records and Statistics, ensuring comprehensive capture of deaths occurring after hospital discharge. For patients who survived beyond day 30, follow-up was administratively truncated, meaning their data were deliberately excluded beyond this predefined time point. This administrative right truncation represents a known and intentional information limit, which is transparently reported in Supplementary Table S1.

The proportion of missing values in our dataset was very low. There were no missing values for key baseline predictors such as age and sex. The SOFA score on day 1 was available for all patients, as it was required for Sepsis-3 diagnosis. Liver function parameters (alanine aminotransferase, aspartate aminotransferase, bilirubin, alkaline phosphatase, and international normalized ratio) were required for the identification of sepsis-associated liver injury within the first seven days after sepsis onset. Using this screening window ensured that relevant values were captured whenever available in clinical practice. Consequently, fewer than 1% of patients had missing liver function parameters within this period and were excluded. Comorbidities had a missing rate of less than 2% and were maybe not missing at random, which was deemed against imputation.

Statistical significance was determined by a p-value less than 0.05, with 95% confidence intervals (CIs) provided for the estimates. All statistical analyses and reporting were conducted in accordance with current recommendations for transparent and robust statistical practices in medical research.^20^^,^^21^ Furthermore, all statistical procedures were conducted using R software (version 3.5.3; The R Foundation for Statistical Computing; http://www.R-project.org).

### Role of funding source

No funding was received. This study was exclusively financed by the authors’ institutional resources. Therefore, the funders had no role in the study design, data collection, data analysis, data interpretation, or writing of the report.

## Results

### Baseline characteristics

The baseline characteristics of septic patients within the MIMIC-IV cohort, stratified by the occurrence of SALI, are presented in Table 1. Patients with SALI were generally younger (63.7 years, ±16.2) compared to those without SALI (65.7 years, ±16.3). While this difference was statistically significant (p < 0.0001), the observed difference of 2 years likely has limited clinical impact. As a result of the selection criteria for identifying SALI, elevated levels of ALT, AST, and total bilirubin were observed in the SALI group compared to patients without SALI. Baseline characteristics of the Amsterdam UMC Cohort are available in Supplementary Table S2.

**Table 1:** Baseline characteristics of septic patients within the MIMIC-IV cohort, stratified by the occurrence of SALI within 7 days after sepsis diagnosis.

	Overall (n = 12,716)	SALI (n = 3235)	No SALI (n = 9481)	p-value
**Demographics**				
Age [years], mean (SD)	65.2 (16.3)	63.7 (16.2)	65.7 (16.3)	<0.0001
Female sex, n (%)	7244 (57)	1855 (57)	5389 (57)	0.62
**Admission type, n (%)**				
Medical	9577 (75)	2599 (80)	6978 (74)	<0.0001
Surgical	3139 (25)	636 (20)	2503 (26)	
**Source of infection, n (%)**				
Pulmonal	5181 (41)	1138 (36)	4043 (43)	<0.0001
Abdominal	1493 (12)	764 (24)	729 (8)	
Urogenital	1786 (14)	337 (10)	1449 (15)	
Other/Unknown	4256 (33)	996 (31)	3260 (34)	
**Medical history, n (%)**				
Myocardial infarction	1857 (15)	379 (12)	1478 (16)	<0.0001
Heart failure	3335 (26)	690 (21)	2645 (28)	<0.0001
Cerebrovascular disease	1702 (13)	256 (8)	1446 (15)	<0.0001
Chronic pulmonary disease	3217 (25)	641 (20)	2576 (27)	<0.0001
Mild/moderate/severe liver disease	0 (0)	0 (0)	0 (0)	1
Diabetes mellitus without complications	2928 (23)	637 (20)	2291 (24)	<0.0001
Diabetes mellitus with complications	1127 (9)	250 (8)	877 (9)	0.0089
Renal disease	2711 (21)	582 (20)	2129 (22)	<0.0001
Malignant diseases	1554 (12)	432 (13)	1122 (12)	0.023
**Clinical parameters**				
SOFA score on day 1, mean (SD)	6.0 (2.9)	7.4 (3.2)	5.5 (2.5)	<0.0001
Septic shock on day 1, n (%)	2287 (198)	1004 (31)	1283 (14)	<0.0001
ICU length of stay [days], median (IQR)	4 [2–7]	4 [2–8]	3 [2–7]	<0.0001
30-Day mortality, n (%)	2069 (16)	807 (25)	1262 (13)	<0.0001
**Laboratory values at ICU admission (first 24 h); mean (SD) or median (IQR)**				
Hemoglobin (g/dL)	10.3 (2.0)	10.0 (2.0)	10.4 (2.0)	<0.0001
Leukocyte count (1000/μL)	11.9 [8.4–16.4]	13.0 [8.6–18.6]	11.7 [8.4–15.8]	<0.0001
Serum creatinine [mg/dL]	1.15 [0.80–1.90]	1.35 [0.90–2.26]	1.10 [0.80–1.75]	<0.0001
Serum lactate [mmol/L]	2.0 [1.4–3.0]	2.4 [1.6–4.2]	1.8 [1.3–2.7]	<0.0001
ALT [U/L]	29 [16–67]	84 [33–289]	22 [14–40]	<0.0001
AST [U/L]	42 [24–98]	116 [51–365]	32 [21–59]	<0.0001
Total bilirubin (μmol/L)	0.7 [0.4–1.3]	1.7 [0.7–3.5]	0.5 [0.4–0.9]	<0.0001
ALP (U/L)	83 [60–127]	151 [91–260]	73 [56–98]	<0.0001
INR	1.30 [1.20–1.60]	1.50 [1.30–1.90]	1.30 [1.15–1.50]	<0.0001

SALI: Sepsis-associated liver injury; MIMIC-IV: Medical Information Mart for Intensive Care-IV database; SD: Standard deviation; IQR: Interquartile range; SOFA: Sequential Organ Failure Assessment score; ICU: Intensive care unit; ALT: Alanine aminotransferase; AST: Aspartate aminotransferase; ALP: Alkaline phosphatase; INR: International normalized ratio.

**Fig. 1:**
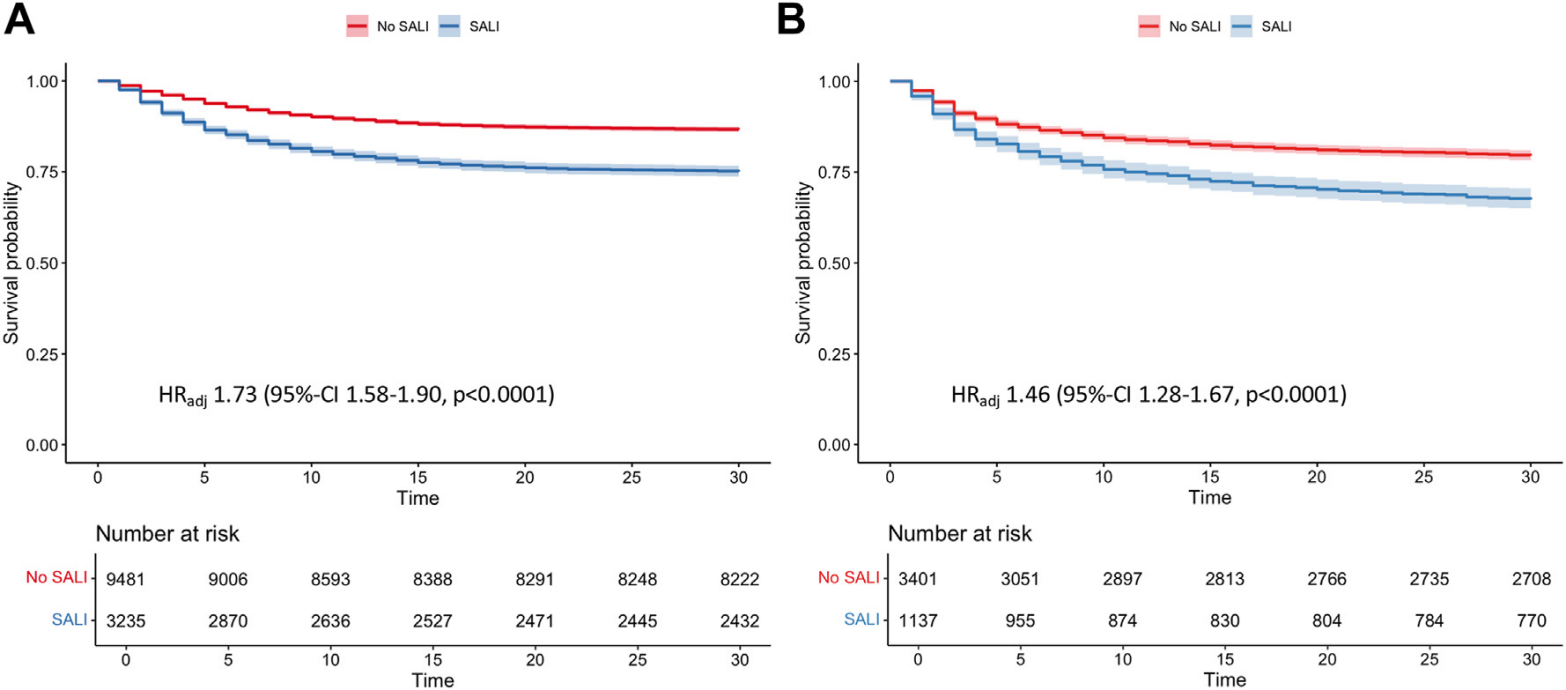
**30-day survival of sepsis patients with and without SALI.** Kaplan–Meier survival curves for patients with SALI in both the MIMIC-IV derivation cohort **(A)** and the Amsterdam UMC validation cohort **(B)**. In both cohorts, patients with SALI exhibited significantly lower 30-day survival probabilities compared to those without SALI, with a HR of 1.73 (95% CI: 1.58–1.90, p < 0.0001) in the MIMIC-IV cohort and an HR of 1.46 (95% CI: 1.28–1.67, p < 0.0001) in the Amsterdam UMC cohort. SALI: Sepsis-associated liver injury; MIMIC-IV: Medical Information Mart for Intensive Care-IV database, HR: Hazard ratio; CI: Confidence Interval.

As depicted in Fig. 1, Kaplan–Meier survival curves showed a clinically meaningful reduction in 30-day survival probabilities among patients with SALI. The hazard ratio (HR) for 30-day mortality in the MIMIC-IV cohort was 1.73 (95% CI: 1.58–1.90, p < 0.0001, Fig. 1a), suggesting a robust association between SALI and increased mortality risk. A similar association was observed in the Amsterdam UMC cohort (HR: 1.46, 95% CI: 1.28–1.67, p < 0.0001, Fig. 1b).

### Stratification according to De Ritis ratio

**Fig. 2:**
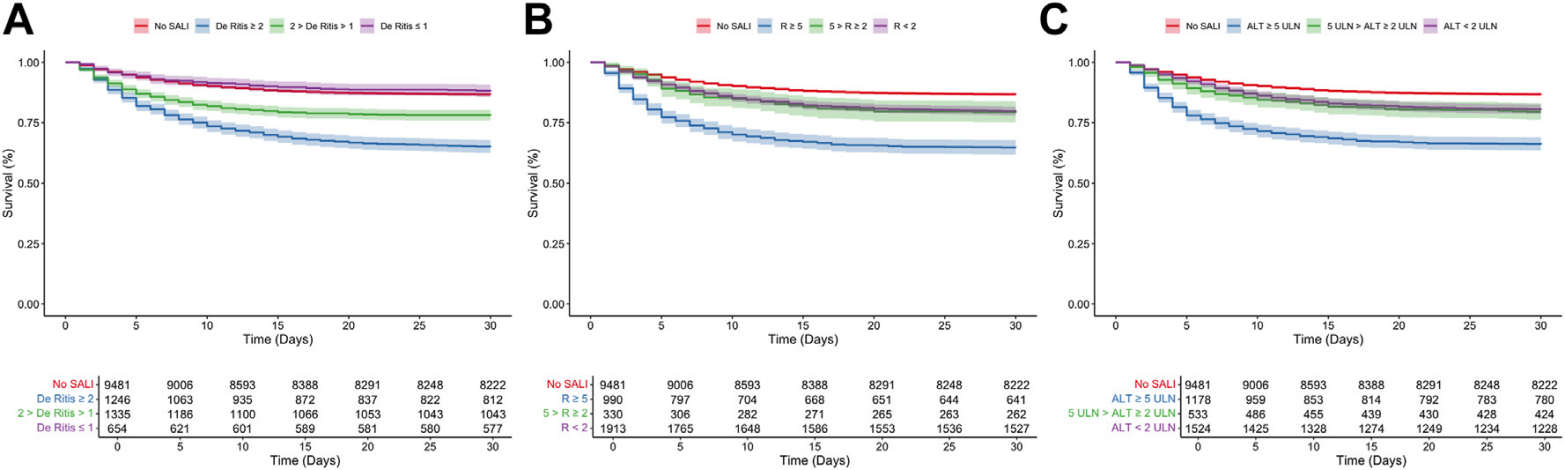
**30-day survival of SALI patients stratified by the De Ritis ratio. (A)** Kaplan–Meier survival curves stratified by the De Ritis ratio show a clear separation in 30-day survival probabilities. Patients with a De Ritis ratio ≥2 had a significantly higher risk of mortality (HR 2.46, 95% CI: 2.18–2.77, p < 0.0001) compared to those with a ratio between 1 and 2 (HR 1.56, 95% CI: 1.37–1.78, p < 0.0001) and those with a ratio ≤1, who did not exhibit an increased mortality risk (HR: 0.86; 95% CI: 0.68–1.09, p = 0.21). **(B)** Stratification by the R-factor identified patients at elevated risk, with those in the R ≥ 5 category showing the highest mortality risk (HR 2.19, 95% CI: 1.93–2.49, p < 0.0001), followed by the 5 > R ≥ 2 group (HR 1.43, 95% CI: 1.12–1.82, p = 0.0045) and the R < 2 group (HR 1.53, 95% CI: 1.36–1.72, p < 0.0001). **(C)** Stratification by ALT concentrations demonstrated increased mortality in patients with ALT levels ≥5 times the ULN (HR 2.16, 95% CI 1.91–2.44, p < 0.0001), with progressively lower risks in the 5 ULN > ALT ≥2 ULN group (HR 1.42, 95% CI: 1.16–1.73, p < 0.0001) and the ALT <2 ULN group (HR 1.41, 95% CI: 1.23–1.60, p < 0.0001). SALI: Sepsis-associated liver injury; HR: Hazard ratio; CI: Confidence Interval; ALT: Alanine aminotransferase; ULN: Upper limit of normal.

Table 2 presents the baseline characteristics of patients with SALI within the MIMIC-IV cohort, stratified by the De Ritis ratio. Patients with a De Ritis ratio ≥2 were generally younger, had higher SOFA scores, and were more likely to present with septic shock compared to those with a De Ritis ratio ≤1. The 30-day mortality rate was highest in the group with a De Ritis ratio ≥2 at 35%, followed by 22% in the group with a De Ritis ratio between 1 and 2, and 12% in the group with a De Ritis ≤1. Kaplan–Meier survival analysis, as illustrated in Fig. 2a, confirmed differences in 30-day survival probabilities across these groups. Specifically, the HR for 30-day mortality (Table 3) was 2.46 (95% CI: 2.18–2.77, p < 0.0001) for patients with a De Ritis ratio ≥2 and 1.56 (95% CI: 1.37–1.78, p < 0.0001) for those with a ratio between 1 and 2. In contrast, for patients with a De Ritis ratio ≤1, no increased mortality risk was observed compared to those without SALI (HR: 0.86; 95% CI: 0.68–1.09, p = 0.21). Further statistical details on the Cox regression analyses, including model fit and performance metrics, are provided in Supplementary Table S3.

**Table 2:** Baseline characteristics of SALI patients within the MIMIC-IV cohort, stratified by De Ritis ratio.

	De Ritis ≥ 2 (n = 1,246)	2 > De Ritis > 1 (n = 1,335)	De Ritis ≤ 1 (n = 654)	p-value
**Demographics**				
Age [years], mean (SD)	65.2 (16.3)	63.7 (16.2)	65.7 (16.3)	<0.0001
Female sex, n (%)	707 (57)	761 (57)	387 (59)	0.24
**Admission type, n (%)**				
Medical	998 (80)	1056 (79)	545 (83)	0.11
Surgical	248 (20)	279 (21)	109 (17)	
**Source of infection, n (%)**				
Pulmonal	448 (36)	466 (35)	224 (34)	<0.0001
Abdominal	239 (19)	331 (25)	194 (30)	
Urogenital	138 (11)	134 (10)	65 (10)	
Other/Unknown	421 (34)	404 (30)	171 (26)	
**Medical history, n (%)**				
Myocardial infarction	140 (11)	152 (12)	87 (13)	0.41
Heart failure	223 (18)	318 (24)	149 (23)	0.0021
Cerebrovascular disease	74 (6)	134 (10)	48 (7)	0.0009
Chronic pulmonary disease	206 (17)	296 (22)	139 (21)	0.0025
Mild/ moderate/ severe liver disease	0 (0)	0 (0)	0 (0)	1
Diabetes mellitus without complications	210 (17)	293 (22)	134 (21)	0.011
Diabetes mellitus with complications	90 (7)	112 (8)	48 (7)	0.57
Renal disease	214 (17)	247 (19)	121 (19)	0.13
Malignant diseases	130 (10)	211 (16)	91 (14)	0.0002
**Clinical parameters**				
SOFA score on day 1, mean (SD)	6.0 (2.9)	7.4 (3.2)	5.5 (2.5)	<0.0001
Septic shock on day 1, n (%)	517 (42)	387 (29)	100 (15)	<0.0001
ICU length of stay [days], median (IQR)	5 [2–10]	4 [2–8]	3 [2–5]	<0.0001
30-Day mortality, n (%)	435 (35)	294 (22)	78 (12)	<0.0001
**Laboratory values at ICU admission (first 24 h); mean (SD) or median (IQR)**				
Hemoglobin [g/dL]	10.3 (2.0)	10.0 (2.0)	10.4 (2.0)	<0.0001
Leukocyte count [1000/μL]	13.0 [8.6–18.6]	13.1 [8.4–18.7]	12.8 [8.9–17.9]	0.0026
Serum creatinine [mg/dL]	1.53 [0.95–2.60]	1.30 [0.87–2.15]	1.20 [0.80–1.90]	<0.0001
Serum lactate [mmol/L]	2.8 [1.8–4.9]	2.4 [1.6–4.0]	2.0 [1.4–2.7]	<0.0001
ALT [U/L]	44 [21–156]	91 [38–317]	191 [86–391]	<0.0001
AST [U/L]	115 [51–433]	116 [47–407]	117 [53–275]	<0.0001
Total bilirubin [μmol/L]	1.9 [0.8–4.0]	1.5 [0.6–3.1]	1.9 [0.7–3.7]	<0.0001
ALP [U/L]	135 [82–236]	158 [95–269]	193 [108–292]	<0.0001
INR	1.60 [1.30–2.10]	1.45 [1.25–1.87]	1.40 [1.20–1.70]	<0.0001

SALI: Sepsis-associated liver injury; MIMIC-IV: Medical Information Mart for Intensive Care-IV database; SD: Standard deviation; IQR: Interquartile range; SOFA: Sequential Organ Failure Assessment score; ICU: Intensive care unit; ALT: Alanine aminotransferase; AST: Aspartate aminotransferase; ALP: Alkaline phosphatase; INR: International normalized ratio.

**Table 3:** Stratification of SALI patients in the MIMIC-IV and UMC Amsterdam cohorts using cox regression analysis.

	MIMIC-IV cohort		UMC Amsterdam cohort	
	Harzard ratio (95% CI)	p-value	Harzard ratio (95% CI)	p-value
De Ritis ratio				
De Ritis ≥2	2.46 (2.18–2.77)	<0.0001	1.78 (1.50–2.12)	<0.0001
2 > De Ritis >1	1.56 (1.37–1.78)	<0.0001	1.30 (1.08–1.56)	0.0044
De Ritis ≤1	0.86 (0.68–1.09)	0.21	1.18 (0.86–1.62)	0.36
R-factor				
R ≥ 5	2.19 (1.93–2.49)	<0.0001	1.97 (1.68–2.31)	<0.0001
5 > R ≥2	1.43 (1.12–1.82)	0.0045	1.25 (0.90–1.72)	0.18
R < 2	1.53 (1.36–1.72)	<0.0001	1.01 (0.82–1.25)	0.92
ALT concentration				
ALT ≥ 5 ULN	2.16 (1.91–2.44)	<0.0001	1.92 (1.65–2.24)	<0.0001
5 ULN > ALT ≥ 2 ULN	1.42 (1.16–1.73)	<0.0001	1.10 (0.80–1.52)	0.55
ALT < 2 ULN	1.41 (1.23–1.60)	<0.0001	0.97 (0.77–1.23)	0.80

SALI: Sepsis-associated liver injury; MIMIC-IV: Medical Information Mart for Intensive Care-IV database; CI: Confidence interval; ALT: Alanine aminotransferase; ULN: Upper limit of normal.

The adequacy of our sample size was assessed based on the precision of key effect estimates. We evaluated the width of the 95% CIs for our primary HR estimates to ensure robust and reliable conclusions. The narrow CIs observed for the key HR (see Fig. 1, Table 3) indicate high precision in our effect estimates, supporting the validity of our findings. This reinforces that the available sample size was sufficient to assess the study objectives.

### Stratification according to R

Supplementary Table S4 presents the baseline characteristics of patients with SALI in the MIMIC-IV cohort, stratified by the R-factor. Patients with an R ≥ 5 were more likely to present with septic shock and had higher SOFA scores compared to those with an R < 2. The 30-day mortality rates were 35% for the R ≥ 5 group, 21% for the group with R between 5 and 2, and 20% for the R < 2 group. Kaplan–Meier survival analysis, as shown in Fig. 2b, demonstrated significant differences in 30-day survival probabilities across these groups. The HR for 30-day mortality (Table 3) were notably elevated: R ≥ 5 had a HR of 2.19 (95% CI: 1.93–2.49, p < 0.0001), R between 5 and 2 was associated with a HR of 1.43 (95% CI: 1.2–1.82, p = 0.0045), and R < 2 had a HR of 1.53 (95% CI: 1.36–1.72, p < 0.0001).

### Stratification according to alanine aminotransferase

Supplementary Table S5 presents the baseline characteristics of patients with SALI within the MIMIC-IV cohort, stratified by ALT concentrations. Patients with ALT levels ≥5 times the ULN exhibited higher SOFA scores, and a higher incidence of septic shock compared to those with ALT <2 times the ULN. The 30-day mortality rate was markedly elevated in the group with ALT ≥5 ULN at 34%, followed by 21% in the group with ALT between 2 and 5 ULN, and 20% in the ALT <2 ULN group. Kaplan–Meier survival analysis, depicted in Fig. 2c, revealed significant differences in 30-day survival probabilities among these groups. Specifically, the HR for 30-day mortality (Table 3) was 2.16 (95% CI: 1.91–2.44, p < 0.0001) for patients with ALT ≥5 ULN, 1.42 (95% CI: 1.16–1.73, p < 0.0001) for those with ALT between 2 and 5 ULN, and 1.41 (95% CI: 1.23–1.60, p < 0.0001) for those with ALT <2 ULN.

### Validation of stratification within Amsterdam UMC cohort

We successfully replicated the findings in the Amsterdam UMC cohort, confirming that the De Ritis ratio serves as a robust prognostic marker with strong discriminatory power, as illustrated in Table 3 and Supplementary Fig. S2a. The stratification methods based on the R-factor (Table 3, Supplementary Fig. S2b) and transaminases (Table 3, Supplementary Fig. S2c) demonstrated similar trends in predictive value to those observed in the derivation cohort. The baseline characteristics of the subgroups resulting from these stratifications are detailed in Supplementary Tables S6–S8.

### Stratification of 30-day mortality risk in SALI patients by De Ritis ratio, infection source and admission type

We examined whether the prognostic performance of the De Ritis ratio differed by sepsis aetiology or admission type. Specifically, we stratified patients by the source of infection into pulmonary (Supplementary Fig. S3a), abdominal (Supplementary Fig. S3b), urogenital (Supplementary Fig. S3c), and other/unknown (Supplementary Fig. S3d), as detailed in Supplementary Table S9. The baseline characteristics of these subgroups are provided in Supplementary Tables S10–S15. Supplementary Fig. S4a and b illustrate the 30-day survival probabilities of SALI patients stratified by De Ritis ratio and admission type (medical and surgical). Our findings indicate that a De Ritis ratio ≥2 consistently identifies patients at high risk of 30-day mortality across all infection sources and admission types (Supplementary Figs. S3 and S4 and Table S9). However, in the intermediate group (De Ritis ratio between 1 and 2), we observed variability: patients with abdominal infections exhibited a more gradual decline in survival compared to those with pulmonary or urogenital infections. This suggests that while the De Ritis ratio remains a robust prognostic marker overall, the underlying aetiology of sepsis may modulate risk predictions, particularly in the intermediate range. In a further analysis, we extended the multivariable Cox regression model to include the source of infection as an additional covariate. The results remained robust, confirming the De Ritis ratio as an independent predictor of 30-day mortality (Supplementary Table S16). Furthermore, Kaplan–Meier curves show that SALI patients with a medical admission and a De Ritis ratio ≥2 had a significantly higher mortality risk (HR 2.52, 95% CI: 2.21–2.87, p < 0.0001; Supplementary Fig. S4a) compared to those with a ratio ≤1, who showed no increased risk (Supplementary Table S17). Patients with a De Ritis ratio between 1 and 2 (2 > De Ritis > 1) had an intermediate mortality risk, more pronounced in medical versus surgical admissions (Supplementary Fig. S4a and b).

## Discussion

Our study provides novel insights into the outcome-related stratification of 30-day mortality in patients with SALI by employing the De Ritis ratio, R-factor, and the extent of ALT serum activity. Among these approaches, the De Ritis ratio proved to be the most effective, demonstrating a strong capacity to distinguish between three risk groups in both the MIMIC-IV derivation cohort and the Amsterdam UMC validation cohort, encompassing the prognosis “good”, “bad” and “ugly”. The De Ritis ratio, which relates AST to ALT, has long been recognized in hepatology for its ability to differentiate between types of liver injury.^9^ However, our study is the first to evaluate its discrimination performance in patients with SALI, demonstrating clear prognostic value. While established sepsis severity scores such as SOFA and APACHE II provide a comprehensive assessment of multi-organ dysfunction, our study takes a different approach by focusing specifically on the liver, recognizing its extraordinary role in sepsis phenotypes.^22^ Our results confirm that SALI represents a distinct sepsis phenotype with an increased risk profile, warranting further investigation beyond global sepsis severity scores. Notably, we demonstrated that a De Ritis ratio ≥1 identifies subphenotypes of SALI patients with significantly higher mortality, whereas those with a ratio <1 do not exhibit worse outcomes. This suggests that specific management adjustments are not necessary—or could even be detrimental—for the latter SALI subphenotype. However, in patients with a De Ritis ratio ≥1, targeted interventions seem warranted, underscoring the need for further research. This highlights the potential to guide personalized management strategies linked to the identified three SALI subphenotypes derived from the De Ritis ratio.^23^^,^^24^

When examining the sepsis subgroups, a difference between surgical sepsis and patients admitted for medical reasons becomes apparent. Surgical sepsis often results from localized infections, such as intra-abdominal or post-operative complications, where the primary driver of organ dysfunction may be an ischemia-reperfusion injury rather than a systemic inflammatory response alone. In contrast, medical sepsis frequently originates from systemic infections like pneumonia or urosepsis, which are more likely to trigger widespread inflammation and multi-organ dysfunction, including hepatic involvement. These differing mechanisms might explain why intermediate De Ritis ratios (i.e., 1–2) are associated with a more pronounced risk increase in medical patients. Patients with a medical admission and a De Ritis ratio of ≥2 have a markedly higher mortality risk, indicating more than double the risk compared to those with a ratio of ≤1, who do not exhibit increased risk, supported by previous findings. Additionally, surgical patients typically undergo extensive perioperative management (e.g., fluid resuscitation), which may mitigate liver dysfunction and its prognostic impact. The relative resilience of surgical patients to moderate liver enzyme alterations could contribute to the observed weaker association. While the De Ritis ratio remains a valuable prognostic marker across both groups, these findings highlight the necessity of contextualizing its interpretation based on sepsis aetiology. Future studies should further explore these differences, potentially incorporating additional markers of hepatic and systemic injury to refine risk stratification in distinct sepsis subgroups.

Although both the R-factor and serum transaminase levels provided valuable insights into the risk associated with SALI outcomes, they did not match the prognostic performance of the De Ritis ratio. Our analysis demonstrated that only the higher categories of the De Ritis ratio, R factor, or ALT were significant discriminators for 30-day mortality in both the derivation and validation cohorts. This suggests a more pronounced hepatic dysfunction, as indicated by elevated bilirubin concentrations and INR values. Another possible explanation is that patients with higher De Ritis ratio (R) categories, elevated R factor, or increased ALT may be particularly susceptible to extrahepatic complications, which could escalate into a harmful multi-organ crosstalk associated with SALI.^23^

What distinguishes the De Ritis ratio from other stratification methods is inclusion of AST, an enzyme that reflects not only hepatocellular injury but also damage in muscle and cardiac tissue.^25^, ^26^, ^27^ This broader scope has allowed the De Ritis ratio to demonstrate strong prognostic performance in sepsis and COVID-19, independent of SALI.^28^^,^^29^ For instance, studies have demonstrated that an elevated De Ritis ratio is linked to poor outcomes in conditions like COVID-19 and trauma, underscoring its broader applicability as a prognostic marker beyond liver-specific conditions.^30^, ^31^, ^32^ The ratio effectively captures the balance between liver-specific injury and systemic sources of AST, correlating strongly with the severity of systemic inflammation and multi-organ dysfunction that are typical in sepsis.^33^^,^^34^ Therefore, the predictive power of the De Ritis ratio is likely rooted in its emphasis on AST, which is also localized within the mitochondria.^25^ Mitochondrial dysfunction, a recognized hallmark of sepsis, is intimately associated with impaired energy metabolism, oxidative stress, and organ failure, all of which significantly contribute to increased mortality in septic patients.^35^^,^^36^ In summary, incorporating AST alongside ALT as the well-known De Ritis ratio positions it as a particularly suitable marker for effectively stratifying risk in patients with SALI. This approach allows for a comprehensive assessment that captures not only hepatocellular damage but also systemic and mitochondrial dysfunctions, which are crucial to the pathophysiology of sepsis and closely linked to increased mortality. Additionally, our data indicate that the magnitude of ALT/AST elevation may not be the most critical factor in risk assessment.^33^ Notably, a De Ritis ratio of 2 or higher was significantly associated with increased mortality risk, which appeared to be driven more by low ALT levels rather than elevated AST (see Table 2). In fact, our findings suggest that transaminase levels alone are not reliable for stratification, despite their current prominence in clinical practice for assessing disease severity. In contrast, the De Ritis ratio appears to offer a more precise and clinically relevant approach being able to reconcile current clinical practice. Furthermore, the De Ritis ratio's simplicity and accessibility make it a proper candidate for straightforward clinical use, particularly in critical care settings where rapid, informed decision-making is essential.

To deepen our understanding of SALI, it seems prudent to continue with in-depth characterization of the distinct subphenotypes delineated by De Ritis ratio stratification. Precise phenotyping is a prerequisite for deeper molecular characterization (endotyping) of high-risk patients with sepsis.^12^ Understanding the unique biological mechanisms underlying the identified (sub-)phenotypes is the crucial next step for developing personalized therapeutic strategies.^23^^,^^24^ However, robust phenotypic identification is essential before endotyping can be pursued. Our study provides a novel framework for this initial step, laying the foundation for future research into the molecular underpinnings of high-risk SALI stratified in the phenotypes with a De Ritis ratio of A) ≤ 1, B) 1–2, and C) ≥ 2. Here, we speculate that in sepsis, the De Ritis ratio >1 most likely reflects systemic inflammation and extrahepatic AST release rather than hepatocellular injury. AST, however, is released from extrahepatic sources (e.g., skeletal muscle, erythrocytes, cardiac tissue) during sepsis-induced hypoxia, hemolysis, or rhabdomyolysis, while ALT remains stable due to its hepatocyte-specific localization.^28^^,^^37^ Therefore, a De Ritis ratio >1 does not necessitate liver-specific treatment modification unless accompanied by rising ALT (indicating new hepatocellular injury) or coagulopathy. Instead, it signals heightened systemic inflammation, guiding prognosis and vigilance for multi-organ dysfunction. In this context, we propose that with a De Ritis ratio greater than 1 and normal ALT, priority should be given to controlling the infection source, with close monitoring for extrahepatic damage such as rhabdomyolysis. In cases where the De Ritis ratio exceeds 2.0, escalation of hemodynamic monitoring and assessment for latent organ failure should be considered. If there is a rise in ALT levels, investigation for secondary hepatotoxic effects, such as those caused by drugs, seems prudent. By dissecting the diverse pathophysiological pathways underlying SALI, we can pave the way for tailored treatment approaches within the framework of precision medicine.

Ultimately, it is important to note that within the framework of EASL 2017 guidelines to differentiate ALI, severe ALI, and ALF we deliberately excluded INR from our SALI definition due to its susceptibility to sepsis-associated coagulopathy and its non-mandatory role in (non-severe) ALI.^8^^,^^38^ To evaluate a potential selection bias, we conducted a secondary analysis using the definition of severe ALI, including loss in function described by an INR >1.5 (Supplementary Fig. S5). The results remained consistent, reinforcing the robustness of the De Ritis ratio across different selection criteria for SALI. This finding underscores its prognostic value and highlights the importance of accounting for sepsis-specific alterations when defining SALI.

This study is subject to several important limitations. The MIMIC-IV and Amsterdam UMC datasets vary in terms of patient management and data collection, which could introduce confounding factors. Here, in particular, the differing documentation regarding the focus of the infection and the type of intensive care unit has limited comparability. This heterogeneity might limit the generalizability of the results to other ICU populations. Some subgroup analyses (e.g., Patients with abdominal source of infection) were based on small sample sizes, leading to widened confidence intervals and potential sparse-data bias, which should be considered when interpreting the results.^39^ However, potential limitations such as unmeasured confounding, cohort-specific variability, and the retrospective study design must be acknowledged, as they may affect the robustness and generalizability of our findings. Besides, hazard ratios are subject to selection bias, as they reflect risk only among those at risk at each time point. This may affect interpretation, especially in heterogeneous cohorts with competing risks.^21^ The study adopts modified Drug-Induced Liver Injury (DILI) criteria to identify SALI patients, which have not been fully validated for SALI. This could result in either under- or over-diagnosis of liver injury within the sepsis context, influencing stratification accuracy and the robustness of our findings. While the study focuses on three stratification methods, other risk stratification approaches (e.g., using biomarkers like Interleukin 6 or procalcitonin) might offer complementary or superior predictive power. Thus, we cannot exclude that other risk stratification methods could substitute for, or even replace, the De Ritis approach introduced in this present study. However, these alternative methods need to be explored in future studies, as they are beyond the scope of this research. The progression of sepsis is dynamic, and liver injury can fluctuate over time. The study focuses on static parameters early in sepsis (first 7 days), which may not fully capture the evolving nature of SALI. Longitudinal studies over an extended timeframe might provide deeper insights into temporal changes in risk and yield a more comprehensive understanding of mortality outcomes associated with liver injury in sepsis. Data mining might be seen as less transparent due to its exploratory nature. Despite achieving high and consistent discrimination performance across all cohorts, our analysis effectively derives the newly introduced mortality risk stratification clusters for SALI through a data-driven approach.

This study identifies the De Ritis ratio as a robust tool for stratifying 30-day mortality risk in SALI. A ratio ≥1 predicts worse outcomes, while ≤1 indicates no increased risk. These findings were validated across two ICU cohorts, underscoring the clinical utility of this subphenotyping approach. Given the heterogeneity of SALI, further research should integrate molecular profiling for endotyping and longitudinal assessments to refine risk prediction. By offering a simple yet powerful prognostic tool, the De Ritis ratio may enable more personalized ICU management of SALI.

## Contributors

Conceptualization: TR, AK, LP, BW, AC, AS, MA; Writing the original draft: BW, LP, TR; Revision of original draft: AW, HN, IT, PE, AC, AS, MA, AK; Data curation: LP, BW, IT, AK, TR; Data analysis: LP, BW, AK, TR; Validation: AW, IT, HN, IT, PE, AC, MA, AS; Supervision: TR, AK, AC, AS, MA; LP, BW, HN, AK, TR had full access to and verified the underlying data. All authors read and approved the final version of the manuscript.

## Data sharing statement

This study utilized publicly available data from the MIMIC-IV (available at https://physionet.org/content/mimiciv) and Amsterdam UMC (available at https://amsterdammedicaldatascience.nl) databases, both of which are accessible to researchers upon meeting their respective access requirements. All study-specific extracted subsets of datasets used and analyzed during this study, are available from the corresponding author upon reasonable request and with publication.

## Declaration of interests

All authors declare that they have no conflicts of interest related to this work.
