# Supplementary material for: Subphenotypes and the De Ritis ratio for mortality risk stratification in sepsis-associated acute liver injury: a retrospective cohort study
Source: eClinicalMedicine. 2025 Mar 27;82:103173. doi: 10.1016/j.eclinm.2025.103173 (PMC11987629; doi:10.1016/j.eclinm.2025.103173)
Supplement: Supplementary Figs. S1–S5 and Tables S1–S17 [file mmc1.pdf]

## Supplementary Appendix

# Subphenotypes and the De Ritis Ratio for Mortality Risk Stratification in Sepsis-Associated Acute Liver Injury: A Retrospective Cohort Study

Lars Palmowski, MD[1,*]; Britta Westhus, MD[1,*]; Andrea Witowski, MD[1]; Hartmuth Nowak, MD[1]; Isabella Traut MD[2]; Ali Canbay, PhD[2]; Andreas Schnitzbauer, PhD[3]; Paul Elbers, MD[4]; Michael Adamzik, PhD[1]; Antonios Katsounas, PhD[2,*]; Tim Rahmel, MD[1,*]

*These authors contributed equally to the work

[1] Department of Anesthesiology, Intensive Care and Pain Therapy, University Hospital Knappschaftskrankenhaus Bochum, In der Schornau 23-25, 44892 Bochum, Germany

[2] Department of Internal Medicine, University Hospital Knappschaftskrankenhaus Bochum, In der Schornau 23-25, 44892 Bochum, Germany

[3] Department of Visceral, Oncological, and Transplant Surgery, University Hospital Knappschaftskrankenhaus Bochum, In der Schornau 23-25, 44892 Bochum, Germany

[4] Department of Intensive Care Medicine, Center for Critical Care Computational Intelligence (C4I), Amsterdam Medical Data Science (AMDS), Amsterdam Cardiovascular Science (ACS), Amsterdam Institute for Infection and Immunity (AII), Amsterdam Public Health (APH), Amsterdam UMC, Vrije Universiteit, De Boelelaan 1117, 1081 HV Amsterdam, Netherlands

**Corresponding author:**

PD Dr. med. Tim Rahmel (Tim.Rahmel@Ruhr-Uni-Bochum.de)

## Supplementary Figures

**<u>Supplementary Figure 1:</u> Selection of SALI cases from MIMIC-IV and Amsterdam UMC databases**

This flowchart illustrates the selection process of SALI cases from the MIMIC-IV (n=73,183 ICU cases) and Amsterdam UMC (n=16,194 ICU cases) databases. Initially, 16,075 and 4,881 sepsis cases were identified using Sepsis-III criteria, yielding 16,075 cases from MIMIC-IV and 4,881 from Amsterdam UMC, respectively. After excluding cases with missing laboratory values (AP, ALT, AST, bilirubin) and pre-existing moderate/severe liver disease, 14,497 cases from MIMIC-IV and 4,538 from Amsterdam UMC remained. From these, 4,063 SALI cases were identified in MIMIC-IV and 1,137 in Amsterdam UMC based on modified DILI criteria. The SALI cases were subsequently stratified for further analysis using the De Ritis ratio, R-factor, and serum transaminase levels.

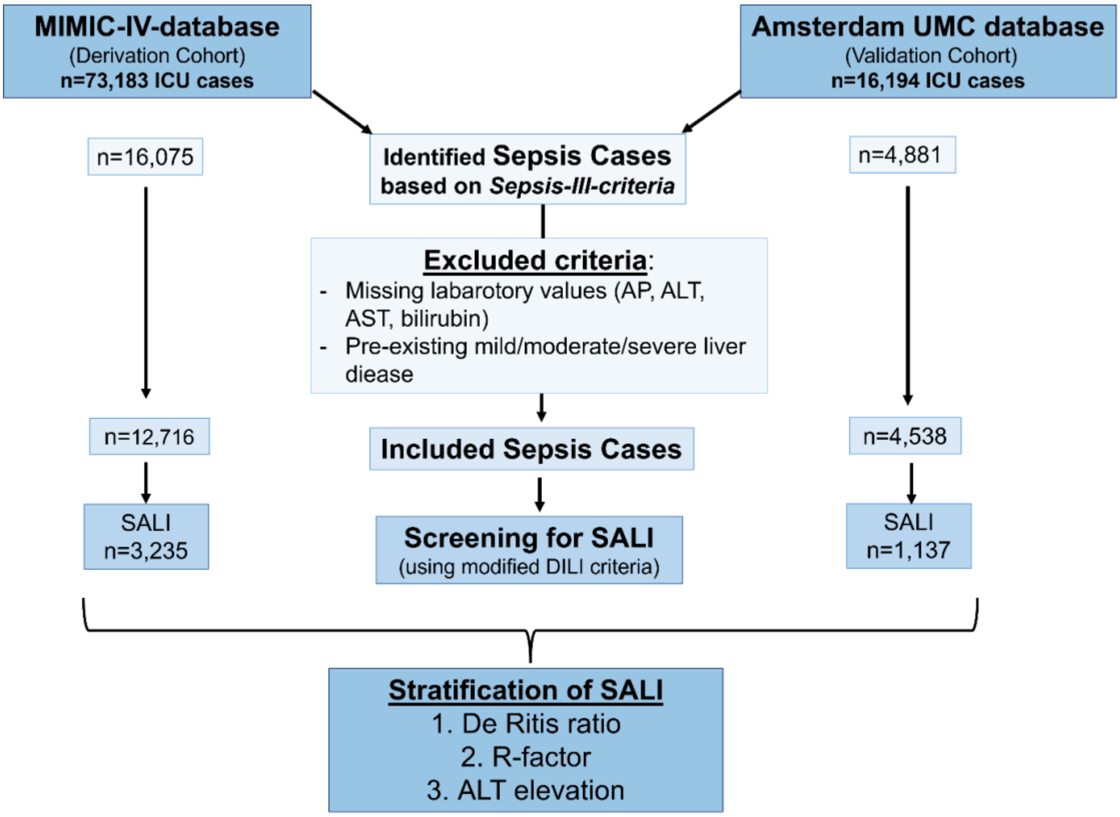

**<u>Supplementary Figure 2:</u> Validation of stratification methods in the Amsterdam UMC cohort**

(A) Kaplan-Meier survival curves, stratified by the De Ritis ratio, reveal distinct 30-day survival probabilities among SALI patients. Those with a De Ritis ratio ≥ 2 had a significantly higher mortality risk (HR 1·78, 95% CI: 1·50-2·12, p<0·0001) compared patients with a ratio ≤ 1, who showed no increased risk relative to patients without SALI. (B) Stratification by the R-factor also identified high-risk patients, with those in the R ≥ 5 category exhibiting the greatest mortality risk (HR 1·97, 95% CI: 1·68-2·31, p<0·0001). (C) Stratification by ALT concentrations demonstrated increased mortality in patients with ALT levels ≥5 times the ULN (HR 1·92, 95% CI: 1·65-2·24, p<0·0001). The shaded area represents the 95% confidence interval around the survival estimates. Although all stratification methods aligned with trends from the derivation cohort, the De Ritis ratio provided the most clear risk group differentiation.

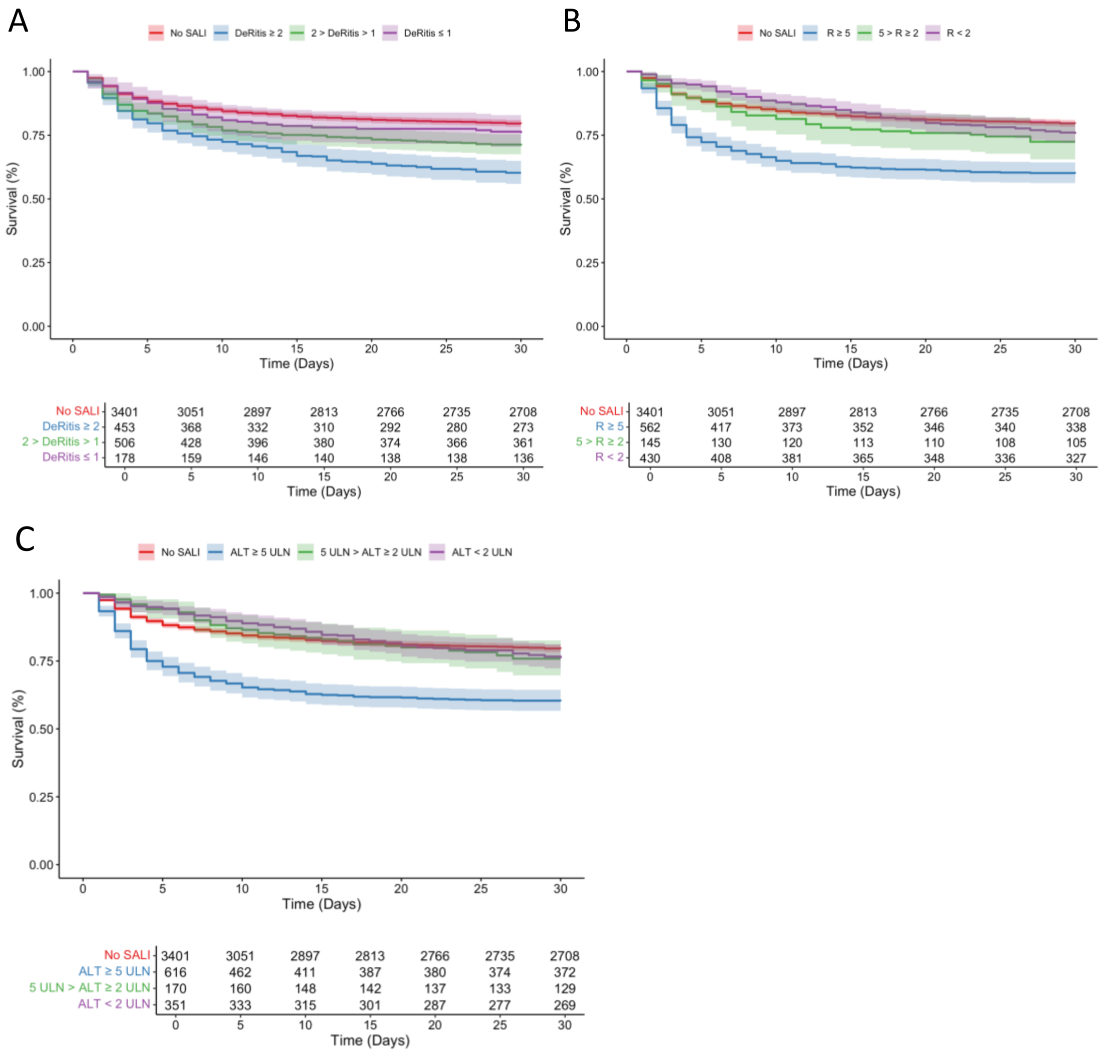

**<u>Supplementary Figure 3:</u> Stratification of SALI Patients in the MIMIC-IV cohort according to the source of infection**

Kaplan-Meier survival curves, stratified by the De Ritis ratio, show distinct 30-day survival probabilities among SALI patients across four infection categories: pulmonary infection (A), abdominal infection (B), urogenital infection (C) and other/unknown infections (D). Patients with a De Ritis ratio ≥ 2 had a significantly higher mortality risk (A: HR 2·45, 95% CI: 2·03-2·95, p<0·0001; B: HR 2·21, 95% CI: 1·59-3·09, p<0·0001; C: HR 3·41, 95% CI: 2·32-4·99, p<0·0001; D: HR 2·55, 95% CI: 2·07-3·14, p<0·0001 ) compared to those with a ratio ≤ 1, who exhibited no increased risk relative to non-SALI patients. Patients with a De Ritis ratio between 1 and 2 (2 > De Ritis > 1) displayed an intermediate mortality risk, with survival probabilities generally lower than those with a ratio ≤ 1 but higher than those with a ratio ≥ 2. Patients with a De Ritis ratio between 1 and 2 (2 > De Ritis > 1) and an abdominal infection show a more gradual decline in survival over 30 days compared to those with pulmonary or urogenital infections or other/unknown infections, indicating a relatively better prognosis within this intermediate risk category. The shaded area represents the 95% confidence interval around the survival estimates.

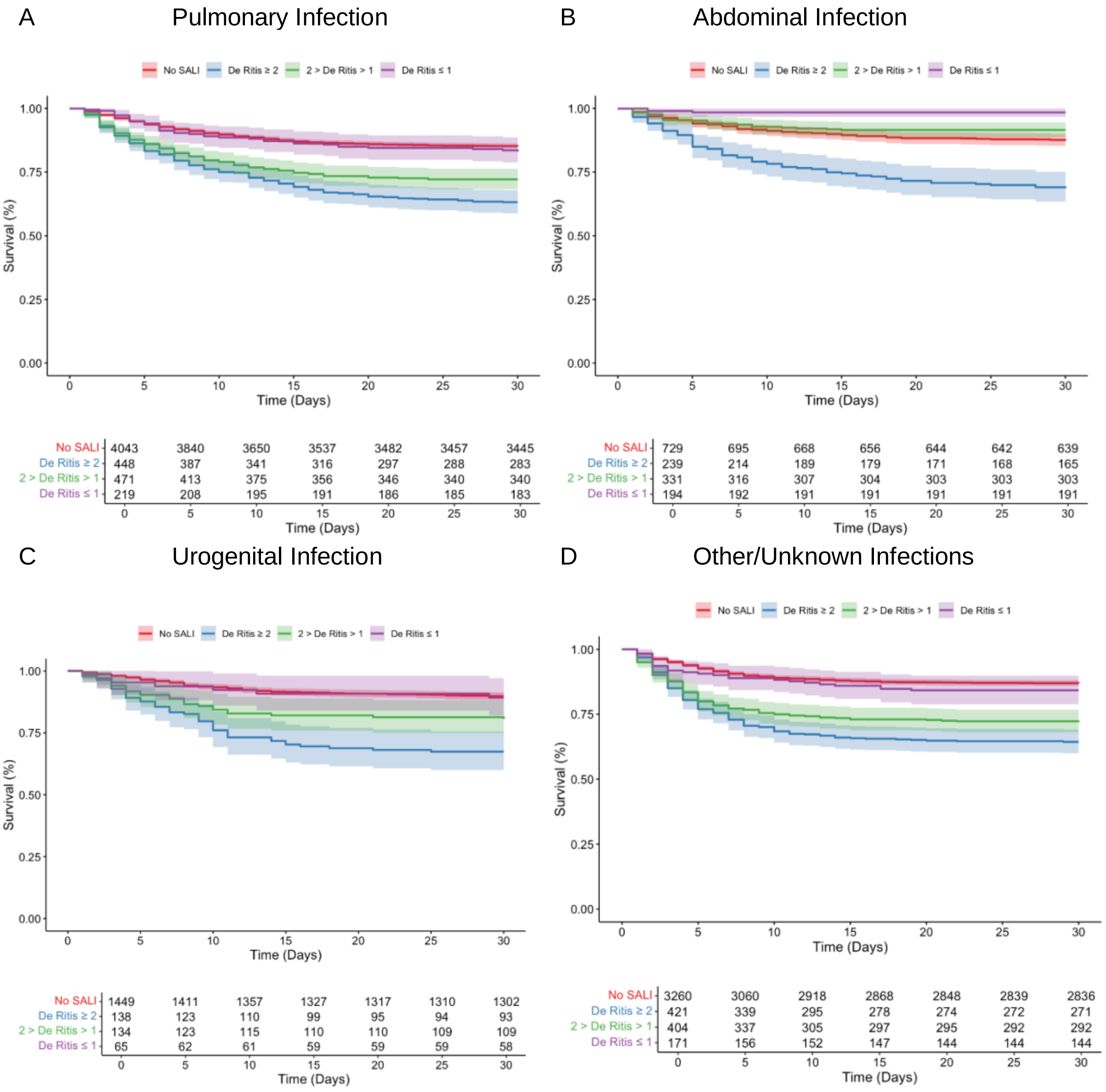

**<u>Supplementary Figure 4:</u> Stratification of SALI patients in the MIMIC-IV Cohort according to the type of admission**

Kaplan-Meier survival curves, stratified by the De Ritis ratio, demonstrate distinct 30-day survival probabilities among SALI patients with medical admission (A) and with surgical admission (B). Those with a De Ritis ratio ≥ 2 had a significantly higher mortality risk (A: HR 2·52, 95% CI: 2·21-2·87, p<0·0001; B: HR 2·19, 95% CI: 1·60-3·00, p<0·0001) compared patients with a ratio ≤ 1, who showed no increased risk relative to patients without SALI. Patients with a ratio between 1 and 2 (2 > De Ritis > 1) had an intermediate mortality risk, reflecting stratification success across different De Ritis ratio thresholds among cases with medical admission (A). The impact of a De Ritis ratio between 1 and 2 (2 > De Ritis > 1) is more substantial in medical patients than in surgical patients, as indicated by a steeper decline in survival probabilities over the 30-day period. The shaded area represents the 95% confidence interval around the survival estimates.

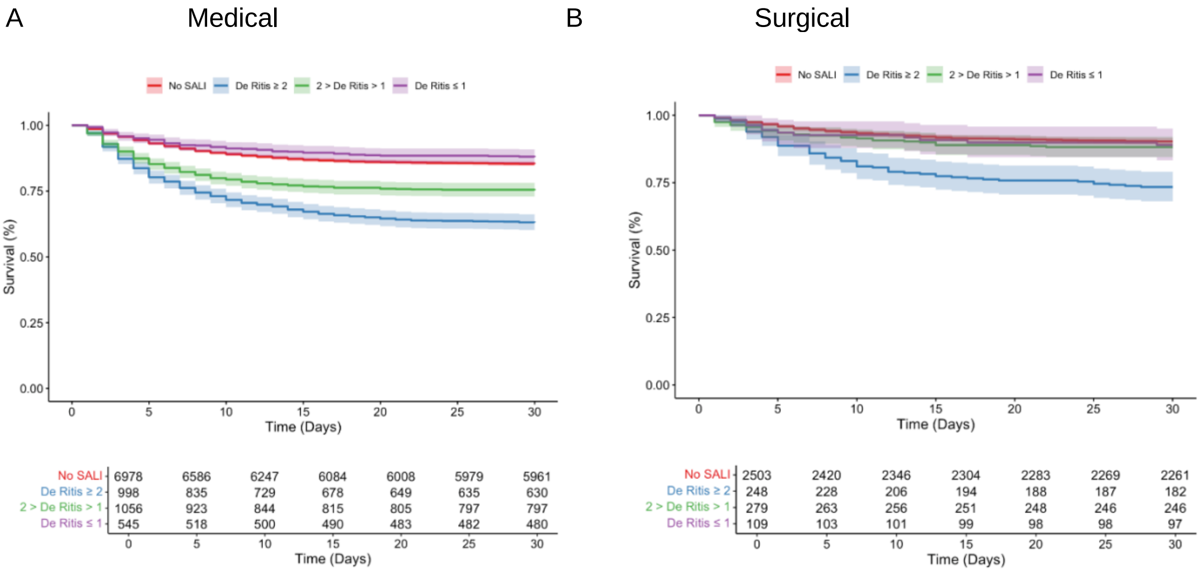

**<u>Supplementary Figure 5:</u> Additional analyses considering an alternative definition of SALI**

Kaplan-Meier survival curves for sepsis patients with and without SALI in both the MIMIC-IV derivation cohort (A) and the Amsterdam UMC validation cohort (B), using the EASL 2017 definitions for severe ALI (AST/ALT > 2 ULN, INR > 1·5, Bilirubin > 2 mg/dL). This more restrictive classification resulted in a marked reduction in the number of cases identified as SALI. However, the overall trend in the adjusted hazard ratios remained consistent. In both cohorts, patients with SALI exhibited significantly lower 30-day survival probabilities compared to those without SALI, with an adjusted hazard ratio (HR) of 2·15 (95% CI: 1·89–2·44, p<0·0001) in the MIMIC-IV cohort and an HR of 1·46 (95% CI: 1·04–1·94, p=0·03) in the Amsterdam UMC cohort.

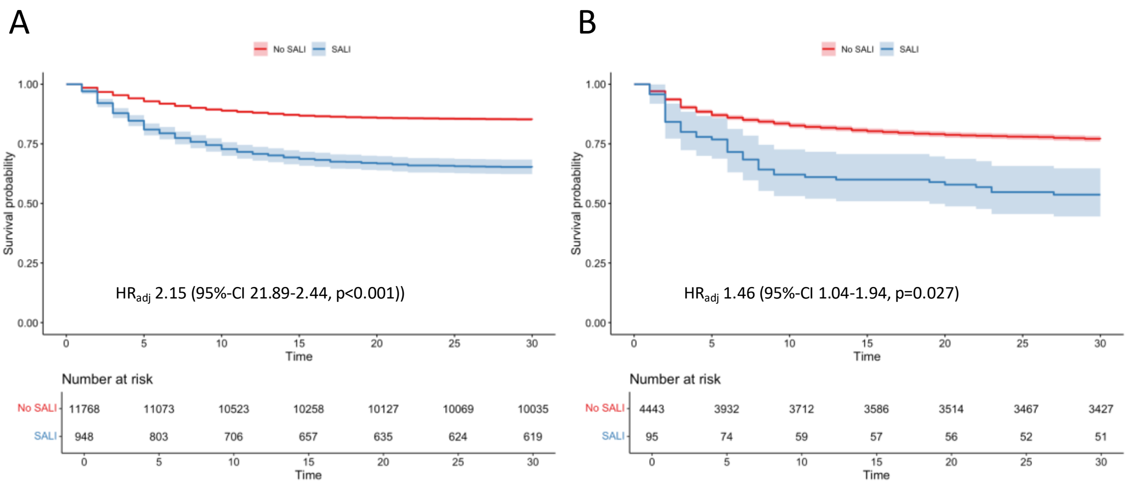


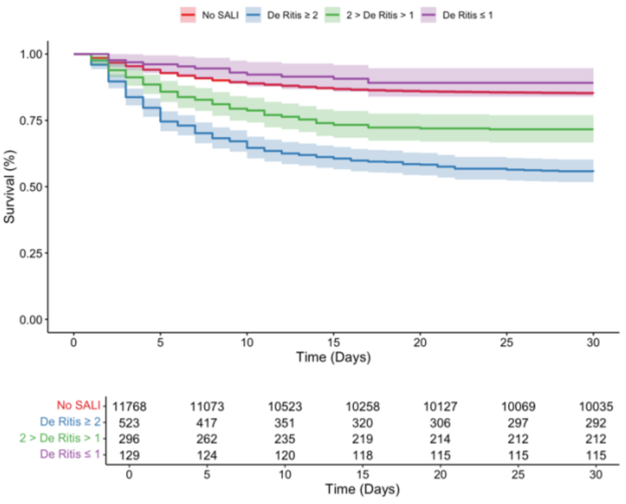


| | | MIMIC-IV Cohort | |
|---|---|---|---|
| | | **Harzard Ratio (95% CI)** | **p-value** |
| **De Ritis ratio** | **De Ritis ≥2** | 2·91 (2·50-3·38) | <0·0001 |
| | **2 > De Ritis >1** | 1·62 (1·29-2·04) | <0·0001 |
| | **De Ritis ≤1** | 0·71 (0·41-1·22) | 0·21 |

# Supplementary Tables

**<u>Supplementary Table 1:</u> Proportion of right-truncated patients at day 30 and median ICU length of stay**

| Cohort | Patients, n | Right-truncated patients at day 30 [%] | Median length of stay (IQR) [days] |
| --- | --- | --- | --- |
| *MIMIC-IV* | 12716 | 83·3% | 9·6 (5·7-17·0) |

MIMIC-IV: Medical Information Mart for Intensive Care-IV database; IQR: Interquartile range

**<u>Supplementary Table 2:</u> Baseline characteristics of septic patients within the Amsterdam UMC cohort, stratified by the occurrence of SALI within 7 days after sepsis diagnosis.**

| | **Overall (n = 4,538)** | **SALI (n = 1,137)** | **No SALI (n = 3,401)** |
|---|---|---|---|
| **Demographics** | | | |
| Age [years], mean (SD) | 62·0 (17·5) | 61·9 (16·9) | 62·1 (17·7) |
| Female sex, n (%) | 2888 (64) | 705 (62) | 2183 (64) |
| **Clinical parameters** | | | |
| SOFA score on day 1, mean (SD) | 6·7 (2·9) | 8·0 (3·2) | 6·3 (2·6) |
| Septic shock on day 1, n (%) | 1688 (37) | 596 (52) | 1092 (32) |
| ICU length of stay [days], median (IQR) | 7 [3 – 15] | 8 [4 – 18] | 6 [3 – 14] |
| 30-Day mortality, n (%) | 1066 (24) | 370 (33) | 696 (21) |
| **Laboratory values at ICU admission (first 24h); mean (SD) or median (IQR)** | | | |
| Hemoglobin (g/dL) | 11·4 (2·0) | 11·1 (2·1) | 11·5 (1·9) |
| Leukocyte count (1000/µL) | 12·5 [9·1 – 16·9] | 12·5 [8·4 – 17·9] | 12·6 [9·3 – 16·5] |
| Serum creatinine (mg/dL) | 1·02 [0·77 – 1·44] | 1·25 [0·88 – 1·84] | 0·96 [0·75– 1·32] |
| Serum lactate (mmol/L) | 2·0 [1·3 – 3·4] | 2·8 [1·6 – 5·2] | 1·8 [1·2 – 2·9] |
| ALT (U/L) | 39 [20 – 103] | 132 [36 – 376] | 32 [18 – 70] |
| AST (U/L) | 60 [30 – 170] | 207 [56 – 606] | 48 [27– 109] |
| Total bilirubin (µmol/L) | 0·6 [0·4 – 0·9] | 0·8 [0·5 – 1·7] | 0·5 [0·4 – 0·8] |
| ALP (U/L) | 68 [50 – 95] | 87 [59 – 155] | 64 [49 – 85] |
| INR | 1·30 [1·15 – 1·54] | 1·47 [1·29 – 1·9] | 1·25 [1·13 – 1·45] |

SALI: Sepsis-associated liver injury; SD: Standard deviation; IQR: Interquartile range; SOFA: Sequential Organ Failure Assessment score; ICU: Intensive care unit; ALT: Alanine aminotransferase; AST: Aspartate aminotransferase; ALP: Alkaline phosphatase; INR: International normalized ratio

**Supplementary Table 3: Model performance metrics for the Cox regression analysis in the MIMIC-IV and UMC cohorts**

| | Performance metrics | SALI vs. No SALI | Stratification of SALI patients according to De Ritis ratio | Stratification of SALI patients according to R-factor | Stratification of SALI patients according to ALT concentration |
|---|---|---|---|---|---|
| MIMIC-IV Cohort | **Concordance** | 0·698 (SE = 0·006) | 0·706 (SE = 0·006) | 0·699 (SE = 0·006) | 0·704 (SE = 0·006) |
| | **Likelihood Ratio Test** | 1066, p < 0·0001 | 1154, p < 0·0001 | 1090, p < 0·0001 | 1152, p < 0·0001 |
| | **Wald Test** | 1144, p < 0·0001 | 1273, p < 0·0001 | 1199, p < 0·0001 | 1253, p < 0·0001 |
| | **Score (Log-rank) Test** | 1185, p < 0·0001 | 1337, p < 0·0001 | 1255, p < 0·0001 | 1312, p < 0·0001 |
| Amsterdam UMC Cohort | **Concordance** | 0·673 (SE = 0·008) | 0·675 (SE = 0·008) | 0·680 (SE = 0·008) | 0·681 (SE = 0·008) |
| | **Likelihood Ratio Test** | 381, p < 0·0001 | 391·1, p < 0·0001 | 413·6, p < 0·0001 | 415, p < 0·0001 |
| | **Wald Test** | 367·9, p < 0·0001 | 382·4, p < 0·0001 | 414·8, p < 0·0001 | 416·8, p < 0·0001 |
| | **Score (Log-rank) Test** | 377·7, p < 0·0001 | 395·2, p < 0·0001 | 428·9, p < 0·0001 | 430·7, p < 0·0001 |

SALI: Sepsis-associated liver injury; MIMIC-IV: Medical Information Mart for Intensive Care-IV database; ALT: Alanine aminotransferase

**Supplementary Table 4:** **Baseline characteristics of SALI patients within the MIMIC-IV cohort, stratified by R-factor**

| | **R ≥ 5** (n = 990) | **5 > R ≥ 2** (n = 330) | **R < 2** (n = 1,913) |
|---|---|---|---|
| **Demographics** | | | |
| Age [years], mean (SD) | 61·6 (17·7) | 64·4 (15·7) | 64·5 (15·3) |
| Female sex, n (%) | 613 (62) | 180 (55) | 1060 (55) |
| **Admission type, n (%)** | | | |
| Medical | 721 (73) | 265 (80) | 1612 (84) |
| Surgical | 269 (27) | 65 (20) | 301 (16) |
| **Source of infection, n (%)** | | | |
| Pulmonal | 374 (38) | 103 (31) | 660 (35) |
| Abdominal | 133 (13) | 96 (29) | 534 (28) |
| Urogenital | 91 (9) | 26 (8) | 220 (12) |
| Other/Unknown | 392 (40) | 105 (32) | 499 (26) |
| **Medical history, n (%)** | | | |
| Myocardial infarction | 124 (13) | 39 (12) | 216 (11) |
| Heart failure | 178 (19) | 68 (20) | 444 (23) |
| Cerebrovascular disease | 68 (7) | 25 (8) | 163 (9) |
| Chronic pulmonary disease | 165 (17) | 71 (22) | 405 (21) |
| Mild/ moderate/ severe liver disease | 0 (0) | 0 (0) | 0 (0) |
| Diabetes mellitus without complications | 149 (15) | 57 (17) | 431 (23) |
| Diabetes mellitus with complications | 49 (5) | 18 (6) | 183 (10) |
| Renal disease | 125 (13) | 47 (14) | 410 (21) |
| Malignant diseases | 62 (6) | 39 (12) | 331 (17) |
| **Clinical parameters** | | | |
| SOFA score on day 1, mean (SD) | 8·2 (3·4) | 7·8 (3·2) | 6·9 (3·1) |
| Septic shock on day 1, n (%) | 517 (52) | 117 (36) | 369 (20) |
| ICU length of stay [days], median (IQR) | 4·5 [2 – 10] | 4 [2 – 7] | 3 [2 – 7] |
| 30-Day mortality, n (%) | 350 (35) | 69 (21) | 388 (20) |
| **Laboratory values at ICU admission (first 24h); mean (SD) or median (IQR)** | | | |
| Hemoglobin (g/dL) | 10·8 (2·2) | 10·5 (2·0) | 9·5 (1·8) |
| Leukocyte count (1000/µL) | 13·7 [9·7 – 18·8] | 13·7 [9·3 – 19·2] | 12·3 [8·1 – 18·2] |
| Serum creatinine (mg/dL) | 1·60 [1·05 –2·40] | 1·30 [0·95 – 2·20] | 1·23 [0·80 – 2·20] |
| Serum lactate (mmol/L) | 3·5 [2·2 – 5·9] | 2·6 [1·7 – 4·3] | 2·0 [1·4 – 2·9] |
| ALT (U/L) | 414 [228 – 954] | 186 [92 – 301] | 43 [23 – 84] |
| AST (U/L) | 555 [271 – 1499] | 219 [119 – 374] | 63 [37 – 115] |
| Total bilirubin (µmol/L) | 1·1 [0·6 – 2·3] | 2·6 [1·0 – 4·2] | 2·0 [0·8 – 4·2] |
| ALP (U/L) | 83 [57 – 122] | 142 [96 – 209] | 223 [137 – 335] |
| INR | 1·58 [1·30 – 2·15] | 1·50 [1·27 – 1·90] | 1·45 [1·25 – 1·80] |

SALI: Sepsis-associated liver injury; MIMIC-IV: Medical Information Mart for Intensive Care-IV database; SD: Standard deviation; IQR: Interquartile range; SOFA: Sequential Organ Failure Assessment score; ICU: Intensive care unit; ALT: Alanine aminotransferase; AST: Aspartate aminotransferase; ALP: Alkaline phosphatase; INR: International normalized ratio

**<u>Supplementary Table 5:</u> Baseline characteristics of SALI patients within the MIMIC-IV cohort, stratified by ALT concentrations**

| | **ALT ≥ 5 ULN** (n = 1,178) | **5 ULN > ALT ≥ 2ULN** (n = 533) | **ALT < 2 ULN** (n = 1,524) |
|---|---|---|---|
| **Demographics** | | | |
| Age [years], mean (SD) | 62·8 (17·3) | 66·1 (15·6) | 63·5 (15·4) |
| Female sex, n (%) | 689 (58) | 315 (59) | 851 (56) |
| **Admission type, n (%)** | | | |
| Medical | 894 (76) | 443 (83) | 1262 (83) |
| Surgical | 284 (24) | 90 (17) | 262 (17) |
| **Source of infection, n (%)** | | | |
| Pulmonal | 437 (37) | 141 (27) | 560 (37) |
| Abdominal | 190 (16) | 211 (40) | 363 (24) |
| Urogenital | 103 (9) | 42 (8) | 192 (13) |
| Other/Unknown | 448 (38) | 139 (26) | 409 (27) |
| **Medical history, n (%)** | | | |
| Myocardial infarction | 147 (13) | 60 (11) | 172 (11) |
| Heart failure | 221 (19) | 106 (20) | 363 (24) |
| Cerebrovascular disease | 82 (7) | 39 (7) | 135 (9) |
| Chronic pulmonary disease | 214 (18) | 88 (17) | 339 (22) |
| Mild/ moderate/ severe liver disease | 0 (0) | 0 (0) | 0 (0) |
| Diabetes mellitus without complications | 189 (16) | 103 (19) | 345 (23) |
| Diabetes mellitus with complications | 62 (5) | 39 (7) | 149 (10) |
| Renal disease | 159 (14) | 90 (17) | 333 (22) |
| Malignant diseases | 91 (7) | 86 (16) | 255 (17) |
| **Clinical parameters** | | | |
| SOFA score on day 1, mean (SD) | 8·0 (3·4) | 7·5 (3·3) | 6·9 (3·0) |
| Septic shock on day 1, n (%) | 555 (47) | 140 (26) | 309 (20) |
| ICU length of stay [days], median (IQR) | 4 [2 – 9] | 3 [2 – 6] | 4 [2 – 8] |
| 30-Day mortality, n (%) | 400 (34) | 109 (21) | 298 (20) |
| **Laboratory values at ICU admission (first 24h); mean (SD) or median (IQR)** | | | |
| Hemoglobin (g/dL) | 10·7 (2·2) | 10·2 (1·9) | 9·5 (1·8) |
| Leukocyte count (1000/µL) | 13·9 [9·8 – 20·0] | 13·1 [8·6 – 18·5] | 11·9 [7·9 – 18·1] |
| Serum creatinine (mg/dL) | 1·60 [1·00 – 2·36] | 1·23 [0·87 – 2·10] | 1·25 [0·80 – 2·23] |
| Serum lactate (mmol/L) | 3·2 [2·0 – 5·7] | 2·3 [1·5 – 4·0] | 2·0 [1·5 – 3·0] |
| ALT (U/L) | 381 [255 – 791] | 134 [103 – 178] | 34 [20 – 53] |
| AST (U/L) | 500 [254 – 1278] | 147 [98 – 229] | 52 [33 – 83] |
| Total bilirubin (µmol/L) | 1·2 [0·6 – 2·7] | 2·8 [1·2– 4·8] | 1·8 [0·7 – 3·5] |
| ALP (U/L) | 102 [64 – 168] | 202 [106 – 336] | 206 [123 – 296] |
| INR | 1·54 [1·30 – 2·13] | 1·44 [1·25 – 1·80] | 1·45 [1·28 – 1·80] |

SALI: Sepsis-associated liver injury; MIMIC-IV: Medical Information Mart for Intensive Care-IV database; ULN: Upper limit of normal; SD: Standard deviation; IQR: Interquartile range; SOFA: Sequential Organ Failure Assessment score; ICU: Intensive care unit; ALT: Alanine aminotransferase; AST: Aspartate aminotransferase; ALP: Alkaline phosphatase; INR: International normalized ratio

**Supplementary Table 6:** **Baseline characteristics of SALI patients within the Amsterdam UMC cohort, stratified by De Ritis ratio**

| | **De Ritis ≥ 2 (n = 453)** | **2 > De Ritis ≥ 1 (n = 506)** | **De Ritis ≤ 1 (n = 178)** |
|---|---|---|---|
| **Demographics** | | | |
| Age [years], mean (SD) | 61·3 (16·2) | 62·9 (17·6) | 60·9 (16·7) |
| Female sex, n (%) | 288 (64) | 301 (60) | 116 (65) |
| **Clinical parameters** | | | |
| SOFA score on day 1, mean (SD) | 8·7 (3·3) | 7·8 (3·0) | 6·9 (3·1) |
| Septic shock on day 1, n (%) | 275 (61) | 263 (52) | 58 (33) |
| ICU length of stay [days], median (IQR) | 10 [4 – 22] | 8 [4 – 17] | 7 [4 – 12] |
| 30-Day mortality, n (%) | 180 (40) | 147 (29) | 43 (24) |
| **Laboratory values at ICU admission (first 24h); mean (SD) or median (IQR)** | | | |
| Hemoglobin (g/dL) | 10·8 (2·0) | 11·3 (2·0) | 11·5 (2·1) |
| Leukocyte count (1000/µL) | 11·8 [7·3 – 17·0] | 13·2 [9·2 – 18·7] | 12·8 [9·5 – 17·4] |
| Serum creatinine (mg/dL) | 1·38 [1·03 – 2·14] | 1·21 [0·89 – 1·74] | 0·96 [0·80 – 1·38] |
| Serum lactate (mmol/L) | 3·2 [1·9 – 6·2] | 2·7 [1·7 – 5·2] | 1·8 [1·2 – 3·0] |
| ALT (U/L) | 72 [28 – 269] | 161 [38 – 423] | 273 [89 – 480] |
| AST (U/L) | 214 [65 – 759] | 221 [47 – 591] | 200 [61 – 316] |
| Total bilirubin (µmol/L) | 0·9 [0·5 – 2·2] | 0·8 [0·5 – 1·5] | 0·7 [0·4 – 1·2] |
| ALP (U/L) | 88 [59 – 157] | 85 [57 – 143] | 95 [65 – 195] |
| INR | 1·55 [1·35 – 1·93] | 1·47 [1·30 – 2·08] | 1·32 [1·20 – 1·52] |

SALI: Sepsis-associated liver injury; SD: Standard deviation; IQR: Interquartile range; SOFA: Sequential Organ Failure Assessment score; ICU: Intensive care unit; ALT: Alanine aminotransferase; AST: Aspartate aminotransferase; ALP: Alkaline phosphatase; INR: International normalized ratio

**Supplementary Table 7: Baseline characteristics of SALI patients within the Amsterdam UMC cohort, stratified by R-factor**

| | **R ≥ 5 (n = 562)** | **5 > R ≥ 2 (n = 145)** | **R < 2 (n = 430)** |
|---|---|---|---|
| **Demographics** | | | |
| Age [years], mean (SD) | 60·9 (17·7) | 60·1 (8·3) | 63·9 (7·5) |
| Female sex, n (%) | 365 (65) | 92 (64) | 248 (58) |
| **Clinical parameters** | | | |
| SOFA score on day 1, mean (SD) | 8·3 (3·1) | 8·3 (3·5) | 7·5 (3·3) |
| Septic shock on day 1, n (%) | 366 (65) | 76 (52) | 154 (36) |
| ICU length of stay [days], median (IQR) | 6 [3 – 14] | 11 [6 – 20] | 11 [6 – 21] |
| 30-Day mortality, n (%) | 225 (40) | 40 (28) | 105 (24) |
| **Laboratory values at ICU admission (first 24h); mean (SD) or median (IQR)** | | | |
| Hemoglobin (g/dL) | 11·6 (2·2) | 11·1 (1·9) | 10·5 (1·7) |
| Leukocyte count (1000/µL) | 12·8 [9·3 – 17·8] | 11·8 [7·5 – 17·8] | 12·1 [7·6 – 18·5] |
| Serum creatinine (mg/dL) | 1·39 [1·00 – 1·96] | 1·27 [0·95 – 1·94] | 1·07 [0·78 – 1·55] |
| Serum lactate (mmol/L) | 4·0 [2·1 – 6·7] | 3·0 [1·6– 4·9] | 1·9 [1·2 – 3·1] |
| ALT (U/L) | 366 [235 – 783] | 88 [49 – 189] | 32 [19 – 57] |
| AST (U/L) | 557 [265 – 1292] | 143 [76 – 361] | 49 [32 – 90] |
| Total bilirubin (µmol/L) | 0·7 [0·4 – 1·3] | 1·0 [0·5 – 2·3] | 1·1 [0·5 – 2·2] |
| ALP (U/L) | 74 [53 – 103] | 79 [48 – 144] | 152 [83 – 252] |
| INR | 1·54 [1·32 – 2·30] | 1·45 [1·29 – 1·97] | 1·40 [1·24 – 1·70] |

SALI: Sepsis-associated liver injury; SD: Standard deviation; IQR: Interquartile range; SOFA: Sequential Organ Failure Assessment score; ICU: Intensive care unit; ALT: Alanine aminotransferase; AST: Aspartate aminotransferase; ALP: Alkaline phosphatase; INR: International normalized ratio

**Supplementary Table 8: Baseline characteristics of SALI patients within the Amsterdam UMC cohort, stratified by ALT concentrations**

| | **ALT ≥ 5 ULN (n = 616)** | **5 ULN > ALT ≥ 2 ULN (n = 170)** | **ALT < 2 ULN (n = 351)** |
|---|---|---|---|
| **Demographics** | | | |
| Age [years], mean (SD) | 61·1 (17·4) | 60·7 (17·6) | 63·9 (15·5) |
| Female sex, n (%) | 397 (65) | 101 (59) | 207 (59) |
| **Clinical parameters** | | | |
| SOFA score on day 1, mean (SD) | 8·3 (3·1) | 7·9 (3·4) | 7·6 (3·3) |
| Septic shock on day 1, n (%) | 386 (63) | 69 (41) | 141 (40) |
| ICU length of stay [days], median (IQR) | 6 [3 – 14] | 11 [6 – 22] | 12 [6 – 22] |
| 30-Day mortality, n (%) | 245 (40) | 41 (24) | 84 (24) |
| **Laboratory values at ICU admission (first 24h); mean (SD) or median (IQR)** | | | |
| Hemoglobin (g/dL) | 11·6 (2·2) | 10·5 (1·7) | 10·5 (1·8) |
| Leukocyte count (1000/µL) | 13·0 [9·4 – 17·9] | 12·0 [7·9 – 18·3] | 11·2 [6·8 – 17·9] |
| Serum creatinine (mg/dL) | 1·37 [0·98 – 1·94] | 1·16 [0·87– 1·81] | 1·11 [0·76 – 1·61] |
| Serum lactate (mmol/L) | 3·7 [2·0 – 6·5] | 2·2 [1·4 – 4·3] | 2·1 [1·4 – 3·3] |
| ALT (U/L) | 355 [200 – 716] | 108 [43 – 154] | 31 [18 – 50] |
| AST (U/L) | 509 [254 – 1155] | 126 [60 – 231] | 47 [31 – 78] |
| Total bilirubin (µmol/L) | 0·7 [0·4 – 1·3] | 1·2 [0·5 – 2·8] | 1·1 [0·6 – 2·2] |
| ALP (U/L) | 77 [56 – 111] | 96 [60 – 213] | 124 [68 – 220] |
| INR | 1·49 [1·31 – 2·27] | 1·40 [1·25 – 1·68] | 1·44 [1·26 – 1·74] |

SALI: Sepsis-associated liver injury; SD: Standard deviation; IQR: Interquartile range; ULN: Upper limit of normal; SOFA: Sequential Organ Failure Assessment score; ICU: Intensive care unit; ALT: Alanine aminotransferase; AST: Aspartate aminotransferase; ALP: Alkaline phosphatase; INR: International normalized ratio

**Supplementary Table 9: Stratification of SALI patients, categorized by source of infection, in the MIMIC-IV cohort using Cox regression analysis**

| Source of infection | De Ritis ratio | Sample size | Harzard ratio (95% CI) | p-value |
|---|---|---|---|---|
| *Pulmonal* | De Ritis ≥2 | 448 | 2·45 (2·03-2·95) | <0·0001 |
| | 2 > De Ritis >1 | 466 | 1·75 (1·44-2·13) | <0·0001 |
| | De Ritis ≤1 | 224 | 1·18 (0·83-1·67) | 0·35 |
| *Abdominal* | De Ritis ≥2 | 239 | 2·21 (1·59-3·09) | <0·0001 |
| | 2 > De Ritis >1 | 331 | 0·67 (0·44-1·04) | 0·072 |
| | De Ritis ≤1 | 194 | 0·04 (0·01-0·32) | 0·0019 |
| *Urogenital* | De Ritis ≥2 | 138 | 3·41 (2·32-4·99) | <0·0001 |
| | 2 > De Ritis >1 | 134 | 1·86 (1·20-2·87) | 0·0051 |
| | De Ritis ≤1 | 65 | 0·98 (0·43-2·23) | 0·96 |
| *Others/ unkown* | De Ritis ≥2 | 421 | 2·55 (2·07-3·14) | <0·0001 |
| | 2 > De Ritis >1 | 404 | 2·01 (1·62-2·50) | <0·0001 |
| | De Ritis ≤1 | 171 | 1·14 (0·76-1·69) | 0·53 |

SALI: Sepsis-associated liver injury; MIMIC-IV: Medical Information Mart for Intensive Care-IV database; CI: Confidence interval

**Supplementary Table 10:** **Baseline characteristics of SALI patients with medical admission within the MIMIC-IV cohort, stratified by De Ritis ratio**

| | **De Ritis ≥ 2** (n = 998) | **2 > De Ritis > 1** (n = 1,056) | **De Ritis ≤ 1** (n = 545) |
|---|---|---|---|
| **Demographics** | | | |
| Age [years], mean (SD) | 60·9 (15·3) | 65·9 (16·0) | 65·7 (16·4) |
| Female sex, n (%) | 570 (57) | 602 (57) | 320 (59) |
| **Source of infection, n (%)** | | | |
| Pulmonal | 362 (36) | 377 (36) | 192 (35) |
| Abdominal | 180 (18) | 241 (23) | 158 (29) |
| Urogenital | 116 (12) | 112 (11) | 55 (10) |
| Other/Unknown | 340 (34) | 326 (31) | 140 (26) |
| **Medical history, n (%)** | | | |
| Myocardial infarction | 114 (11) | 125 (12) | 78 (14) |
| Heart failure | 186 (19) | 272 (26) | 134 (25) |
| Cerebrovascular disease | 50 (5) | 112 (11) | 42 (8) |
| Chronic pulmonary disease | 156 (16) | 250 (24) | 113 (21) |
| Mild/ moderate/ severe liver disease | 0 (0) | 0 (0) | 0 (0) |
| Diabetes mellitus without complications | 169 (17) | 250 (24) | 115 (21) |
| Diabetes mellitus with complications | 75 (8) | 99 (9) | 44 (8) |
| Renal disease | 167 (17) | 205 (19) | 106 (19) |
| Malignant diseases | 111 (11) | 179 (17) | 77 (14) |
| **Clinical Parameters** | | | |
| SOFA score on day 1, mean (SD) | 8·4 (3·5) | 7·3 (3·1) | 6·4 (2·9) |
| Septic Shock on day 1, n (%) | 391 (39) | 306 (29) | 80 (15) |
| ICU length of stay [days], median (IQR) | 4 [2 – 9] | 3 [2 – 7] | 3 [1 – 5] |
| 30-Day mortality, n (%) | 369 (37) | 261 (25) | 66 (12) |
| **Laboratory values at ICU admission (first 24h); mean (SD) or median (IQR)** | | | |
| Hemoglobin (g/dL) | 9·6 (1·9) | 10·1 (2·1) | 10·6 (2·1) |
| Leukocyte count (1000/µL) | 13·3 [8·8 – 19·0] | 13·1 [8·2 – 19·0] | 12·8 [8·8 – 18·0] |
| Serum creatinine (mg/dL) | 1·60 [0·95 – 2·75] | 1·35 [0·90 – 2·30] | 1·20 [0·80 – 2·00] |
| Serum lactate (mmol/L) | 2·6 [1·7 – 4·6] | 2·3 [1·6 – 4·0] | 1·9 [1·4 – 2·6] |
| ALT (U/L) | 42 [21 – 131] | 87 [37 – 284] | 178 [80 – 357] |
| AST (U/L) | 111 [52 – 392] | 107 [46 – 370] | 109 [52 – 257] |
| Total bilirubin (µmol/L) | 2·0 [0·8 – 4·5] | 1·4 [0·6 – 3·1] | 2·0 [0·7 – 3·9] |
| ALP (U/L) | 146 [93 – 244] | 168 [103 – 275] | 199 [117 – 289] |
| INR | 1·62 [1·33 – 2·15] | 1·47 [1·27 – 1·92] | 1·40 [1·20 – 1·70] |

SALI: Sepsis-associated liver injury; MIMIC-IV: Medical Information Mart for Intensive Care-IV database; SD: Standard deviation; IQR: Interquartile range; SOFA: Sequential Organ Failure Assessment score; ICU: Intensive care unit; ALT: Alanine aminotransferase; AST: Aspartate aminotransferase; ALP: Alkaline phosphatase; INR: International normalized ratio

**<u>Supplementary Table 11:</u> Baseline characteristics of SALI patients with surgical admission within the MIMIC-IV cohort, stratified by De Ritis ratio**

| | **De Ritis ≥ 2** (n = 248) | **2 > De Ritis > 1** (n = 279) | **De Ritis ≤ 1** (n = 109) |
|---|---|---|---|
| **Demographics** | | | |
| Age [years], mean (SD) | 63·7 (15·4) | 62·8 (16·9) | 59·1 (19·1) |
| Female sex, n (%) | 137 (55) | 159 (57) | 67 (61) |
| **Source of infection, n (%)** | | | |
| Pulmonal | 86 (35) | 89 (32) | 32 (29) |
| Abdominal | 59 (24) | 90 (32) | 36 (33) |
| Urogenital | 22 (9) | 22 (8) | 10 (9) |
| Other/Unknown | 81 (33) | 78 (28) | 31 (28) |
| **Medical history, n (%)** | | | |
| Myocardial infarction | 26 (10) | 27 (10) | 9 (8) |
| Heart failure | 37 (15) | 46 (16) | 15 (14) |
| Cerebrovascular disease | 24 (10) | 22 (8) | 6 (6) |
| Chronic pulmonary disease | 50 (20) | 46 (16) | 26 (24) |
| Mild/ moderate/ severe liver disease | 0 (0) | 0 (0) | 0 (0) |
| Diabetes mellitus without complications | 41 (17) | 43 (15) | 19 (17) |
| Diabetes mellitus with complications | 15 (6) | 13 (5) | 4 (4) |
| Renal disease | 47 (19) | 42 (15) | 15 (14) |
| Malignant diseases | 19 (8) | 32 (11) | 14 (13) |
| **Clinical Parameters** | | | |
| SOFA score on day 1, mean (SD) | 7·5 (3·1) | 6·6 (3·0) | 5·8 (2·6) |
| Septic shock on day 1, n (%) | 126 (51) | 81 (29) | 20 (18) |
| ICU length of stay [days], median (IQR) | 8 [4 – 16] | 5 [2 – 11] | 4 [2 – 7] |
| 30-Day mortality, n (%) | 66 (27) | 33 (12) | 12 (11) |
| **Laboratory values at ICU admission (first 24h); mean (SD) or median (IQR)** | | | |
| Hemoglobin (g/dL) | 9·9 (1·7) | 10·2 (1·8) | 10·6 (2·1) |
| Leukocyte count (1000/µL) | 12·4 [8·25 – 16·9] | 13·1 [9·8 – 18·3] | 13·0 [9·7 – 17·2] |
| Serum creatinine (mg/dL) | 1·33 [0·95 – 1·99] | 1·13 [0·77 – 1·67] | 1·00 [0·80 – 1·60] |
| Serum lactate (mmol/L) | 3·5 [2·1 – 5·6] | 2·50 [1·60 – 1·05] | 2·3 [1·4 – 3·2] |
| ALT (U/L) | 52 [22 – 228] | 151 [41 – 372] | 331 [108 – 613] |
| AST (U/L) | 153 [50 – 511] | 185 [54 – 513] | 209 [64 – 453] |
| Total bilirubin (µmol/L) | 1·4 [0·8 – 2·7] | 1·6 [0·7 – 3·1] | 1·4 [0·7 – 2·9] |
| ALP (U/L) | 78 [47 – 153] | 112 [63 – 225] | 130 [70 – 304] |
| INR | 1·49 [1·28 – 1·77] | 1·40 [1·25 – 1·69] | 1·30 [1·20 – 1·63] |

SALI: Sepsis-associated liver injury; MIMIC-IV: Medical Information Mart for Intensive Care-IV database; SD: Standard deviation; IQR: Interquartile range; SOFA: Sequential Organ Failure Assessment score; ICU: Intensive care unit; ALT: Alanine aminotransferase; AST: Aspartate aminotransferase; ALP: Alkaline phosphatase; INR: International normalized ratio

**Supplementary Table 12:** **Baseline characteristics of SALI patients with pulmonary source of infection within the MIMIC-IV cohort, stratified by De Ritis ratio**

| | **De Ritis ≥ 2** (n = 448) | **2 > De Ritis > 1** (n = 466) | **De Ritis ≤ 1** (n = 224) |
|---|---|---|---|
| **Demographics** | | | |
| Age [years], mean (SD) | 61·1 (15·3) | 64·9 (15·8) | 62·8 (16·8) |
| Female sex, n (%) | 256 (57) | 265 (57) | 129 (58) |
| **Admission type, n (%)** | | | |
| Medical | 362 (80) | 377 (81) | 192 (86) |
| Surgical | 86 (19) | 89 (19) | 32 (14) |
| **Medical history, n (%)** | | | |
| Myocardial infarction | 45 (10) | 54 (12) | 35 (16) |
| Heart failure | 81 (18) | 126 (27) | 60 (27) |
| Cerebrovascular disease | 19 (4) | 45 (10) | 20 (9) |
| Chronic pulmonary disease | 88 (20) | 143 (31) | 61 (21) |
| Mild/ moderate/ severe liver disease | 0 (0) | 0 (0) | 0 (0) |
| Diabetes mellitus without complications | 68 (15) | 102 (22) | 40 (18) |
| Diabetes mellitus with complications | 33 (7) | 41 (9) | 16 (7) |
| Renal disease | 83 (19) | 92 (20) | 36 (16) |
| Malignant diseases | 51 (11) | 66 (14) | 29 (13) |
| **Clinical Parameters** | | | |
| SOFA score on day 1, mean (SD) | 8·1 (3·4) | 7·3 (2·9) | 6·2 (2·9) |
| Septic shock on day 1, n (%) | 204 (46) | 140 (30) | 36 (16) |
| ICU length of stay [days], median (IQR) | 6 [3 – 14] | 6 [2 – 11] | 4 [2 – 7] |
| 30-Day mortality, n (%) | 165 (37) | 127 (27) | 41 (18) |
| **Laboratory values at ICU admission (first 24h); mean (SD) or median (IQR)** | | | |
| Hemoglobin (g/dL) | 9·7 (1·7) | 10·0 (2·1) | 10·5 (2·2) |
| Leukocyte count (1000/µL) | 12·8 [8·5 – 18·7] | 13·2 [8·4 – 18·6] | 12·3 [8·8 – 17·6] |
| Serum creatinine (mg/dL) | 1·53 [0·90 – 2·38] | 1·38 [0·94 – 2·25] | 1·13 [0·80 – 1·73] |
| Serum lactate (mmol/L) | 2·7 [1·8 – 4·6] | 2·2 [1·5 – 3·8] | 1·9 [1·4 – 2·7] |
| ALT (U/L) | 42 [21 – 151] | 83 [33 – 345] | 227 [73 – 450] |
| AST (U/L) | 115 [52 – 422] | 106 [42 – 466] | 115 [48 – 320] |
| Total bilirubin (µmol/L) | 1·6 [0·8 – 3·4] | 1·0 [0·5 – 2·1] | 0·9 [0·5 – 2·3] |
| ALP (U/L) | 131 [79 – 217] | 137 [90 – 233] | 173 [85 – 263] |
| INR | 1·60 [1·30 – 2·00] | 1·43 [1·22 – 1·90] | 1·40 [1·20 – 1·70] |

SALI: Sepsis-associated liver injury; MIMIC-IV: Medical Information Mart for Intensive Care-IV database; SD: Standard deviation; IQR: Interquartile range; SOFA: Sequential Organ Failure Assessment score; ICU: Intensive care unit; ALT: Alanine aminotransferase; AST: Aspartate aminotransferase; ALP: Alkaline phosphatase; INR: International normalized ratio

**<u>Supplementary Table 13:</u> Baseline characteristics of SALI patients with abdominal source of infection within the MIMIC-IV cohort, stratified by De Ritis ratio**

| | **De Ritis ≥ 2** (n = 239) | **2 > De Ritis > 1** (n = 331) | **De Ritis ≤ 1** (n = 194) |
|---|---|---|---|
| **Demographics** | | | |
| Age [years], mean (SD) | 65·2 (13·8) | 69·7 (14·5) | 69·2 (14·8) |
| Female sex, n (%) | 131 (55) | 188 (57) | 116 (60) |
| **Admission type, n (%)** | | | |
| Medical | 180 (75) | 241 (73) | 158 (83) |
| Surgical | 59 (25) | 90 (27) | 36 (81) |
| **Medical history, n (%)** | | | |
| Myocardial infarction | 33 (14) | 33 (10) | 23 (12) |
| Heart failure | 45 (19) | 69 (21) | 40 (21) |
| Cerebrovascular disease | 20 (8) | 30 (9) | 12 (6) |
| Chronic pulmonary disease | 50 (21) | 82 (25) | 47 (24) |
| Mild/ moderate/ severe liver disease | 0 (0) | 0 (0) | 0 (0) |
| Diabetes mellitus without complications | 58 (24) | 84 (25) | 52 (27) |
| Diabetes mellitus with complications | 18 (8) | 16 (5) | 10 (5) |
| Renal disease | 44 (18) | 55 (17) | 40 (21) |
| Malignant diseases | 36 (15) | 82 (25) | 36 (19) |
| **Clinical Parameters** | | | |
| SOFA score on day 1, mean (SD) | 8·3 (3·4) | 6·8 (3·1) | 6·3 (2·6) |
| Septic shock on day 1, n (%) | 86 (36) | 73 (22) | 23 (12) |
| ICU length of stay [days], median (IQR) | 5 [2 – 11] | 3 [2 – 5] | 2 [2 – 3] |
| 30-Day mortality, n (%) | 75 (31) | 28 (8) | 3 (2) |
| **Laboratory values at ICU admission (first 24h); mean (SD) or median (IQR)** | | | |
| Hemoglobin (g/dL) | 9·6 (1·9) | 9·9 (1·7) | 10·9 (1·8) |
| Leukocyte count (1000/µL) | 14·2 [9·5 – 19·3] | 14·0 [9·1 – 20·6] | 13·8 [9·6 – 19·0] |
| Serum creatinine (mg/dL) | 1·54 [1·05 – 2·63] | 1·08 [0·20 – 1·60] | 1·10 [0·80 – 1·73] |
| Serum lactate (mmol/L) | 2·7 [1·8 – 4·5] | 2·3 [1·6 – 3·5] | 1·6 [1·1 – 2·6] |
| ALT (U/L) | 44 [22 – 113] | 85 [47 – 181] | 186 [115 – 345] |
| AST (U/L) | 108 [51 – 343] | 102 [58 – 220] | 110 [67 – 224] |
| Total bilirubin (µmol/L) | 2·6 [1·2 – 5·1] | 3·2 [1·9 – 5·8] | 3·7 [2·3 – 5·7] |
| ALP (U/L) | 188 [99 – 345] | 239 [145 – 386] | 216 [144 – 347] |
| INR | 1·60 [1·30 – 2·15] | 1·40 [1·30 – 1·70] | 1·35 [1·20 – 1·70] |

SALI: Sepsis-associated liver injury; MIMIC-IV: Medical Information Mart for Intensive Care-IV database; SD: Standard deviation; IQR: Interquartile range; SOFA: Sequential Organ Failure Assessment score; ICU: Intensive care unit; ALT: Alanine aminotransferase; AST: Aspartate aminotransferase; ALP: Alkaline phosphatase; INR: International normalized ratio

**Supplementary Table 14: Baseline characteristics of SALI patients with urogenital source of infection within the MIMIC-IV cohort, stratified by De Ritis ratio**

| | **De Ritis ≥ 2** (n = 138) | **2 > De Ritis > 1** (n = 134) | **De Ritis ≤ 1** (n = 65) |
|---|---|---|---|
| **Demographics** | | | |
| Age [years], mean (SD) | 64·1 (14·4) | 66·9 (17·3) | 65·7 (16·3) |
| Female sex, n (%) | 58 (42) | 61 (46) | 35 (54) |
| **Admission type, n (%)** | | | |
| Medical | 116 (84) | 112 (84) | 55 (85) |
| Surgical | 22 (16) | 22 (16) | 10 (15) |
| **Medical history, n (%)** | | | |
| Myocardial infarction | 18 (13) | 16 (12) | 12 (18) |
| Heart failure | 30 (22) | 31 (23) | 18 (35) |
| Cerebrovascular disease | 11 (8) | 19 (14) | 4 (6) |
| Chronic pulmonary disease | 19 (14) | 19 (14) | 14 (22) |
| Mild/ moderate/ severe liver disease | 0 (0) | 0 (0) | 0 (0) |
| Diabetes mellitus without complications | 25 (18) | 23 (17) | 10 (15) |
| Diabetes mellitus with complications | 12 (9) | 12 (9) | 9 (14) |
| Renal disease | 23 (17) | 26 (19) | 14 (22) |
| Malignant diseases | 15 (11) | 12 (9) | 7 (11) |
| **Clinical Parameters** | | | |
| SOFA score on day 1, mean (SD) | 7·8 (3·6) | 6·9 (3·1) | 6·5 (3·2) |
| Septic shock on day 1, n (%) | 44 (32) | 38 (28) | 12 (18) |
| ICU length of stay [days], median (IQR) | 4 [2 – 9] | 4 [2 – 7] | 3 [2 – 5] |
| 30-Day mortality, n (%) | 45 (33) | 26 (19) | 7 (11) |
| **Laboratory values at ICU admission (first 24h); mean (SD) or median (IQR)** | | | |
| Hemoglobin (g/dL) | 9·6 (1·9) | 10·3 (2·0) | 9·6 (1·9) |
| Leukocyte count (1000/µL) | 13·0 [8·9 – 19·9] | 12·3 [7·6 – 18·5] | 13·1 [8·6 – 19·0] |
| Serum creatinine (mg/dL) | 1·43 [0·80 – 2·60] | 1·40 [0·87 – 2·40] | 1·80 [1·03 – 2·53] |
| Serum lactate (mmol/L) | 2·3 [1·6 – 4·3] | 2·7 [1·5 – 4·3] | 2·1 [1·6 – 2·7] |
| ALT (U/L) | 32 [17 – 74] | 69 [41 – 401] | 151 [71 – 461] |
| AST (U/L) | 101 [43 – 237] | 87 [41 – 401] | 91 [39 – 281] |
| Total bilirubin (µmol/L) | 1·7 [0·8 – 3·8] | 1·2 [0·6 – 2·4] | 1·0 [0·6 – 1·9] |
| ALP (U/L) | 137 [91 – 213] | 144 [86 – 244] | 200 [97 – 296] |
| INR | 1·66 [1·40 – 2·14] | 1·45 [1·30 – 1·90] | 1·40 [1·22 – 1·85] |

SALI: Sepsis-associated liver injury; MIMIC-IV: Medical Information Mart for Intensive Care-IV database; SD: Standard deviation; IQR: Interquartile range; SOFA: Sequential Organ Failure Assessment score; ICU: Intensive care unit; ALT: Alanine aminotransferase; AST: Aspartate aminotransferase; ALP: Alkaline phosphatase; INR: International normalized ratio

**<u>Supplementary Table 15:</u> Baseline characteristics of SALI patients with other/unknown source of infection within the MIMIC-IV cohort, stratified by De Ritis ratio**

| | **De Ritis ≥ 2** (n = 421) | **2 > De Ritis > 1** (n = 404) | **De Ritis ≤ 1** (n = 171) |
|---|---|---|---|
| **Demographics** | | | |
| Age [years], mean (SD) | 59·0 (16·1) | 61·4 (16·8) | 61·3 (18·8) |
| Female sex, n (%) | 262 (62) | 247 (61) | 107 (63) |
| **Admission type, n (%)** | | | |
| Medical | 340 (81) | 326 (81) | 140 (82) |
| Surgical | 81 (19) | 78 (19) | 31 (18) |
| **Medical history, n (%)** | | | |
| Myocardial infarction | 44 (10) | 49 (12) | 17 (10) |
| Heart failure | 67 (16) | 92 (23) | 31 (18) |
| Cerebrovascular disease | 24 (6) | 40 (10) | 12 (7) |
| Chronic pulmonary disease | 49 (12) | 52 (14) | 17 (10) |
| Mild/ moderate/ severe liver disease | 0 (0) | 0 (0) | 0 (0) |
| Diabetes mellitus without complications | 59 (14) | 84 (21) | 32 (19) |
| Diabetes mellitus with complications | 27 (6) | 43 (11) | 13 (8) |
| Renal disease | 64 (15) | 74 (18) | 31 (18) |
| Malignant diseases | 28 (7) | 51 (13) | 19 (11) |
| **Clinical Parameters** | | | |
| SOFA score on day 1, mean (SD) | 8·3 (3·3) | 7·4 (3·2) | 6·4 (2·8) |
| Septic shock on day 1, n (%) | 183 (43) | 136 (34) | 29 (17) |
| ICU length of stay [days], median (IQR) | 4 [2 – 7] | 3 [2 – 6] | 3 [1 – 5] |
| 30-Day mortality, n (%) | 150 (36) | 113 (28) | 27 (16) |
| **Laboratory values at ICU admission (first 24h); mean (SD) or median (IQR)** | | | |
| Hemoglobin (g/dL) | 9·7 (2·0) | 10·3 (2·1) | 10·7 (2·3) |
| Leukocyte count (1000/µL) | 12·2 [8·3 – 17·3] | 12·3 [8·4 – 17·8] | 11·4 [8·7 – 16·5] |
| Serum creatinine (mg/dL) | 1·60 [1·00 – 2·72] | 1·40 [0·90 – 2·35] | 1·20 [0·80 – 1·95] |
| Serum lactate (mmol/L) | 3·1 [1·8 – 5·5] | 2·6 [1·7 – 4·5] | 2·1 [1·5 – 3·1] |
| ALT (U/L) | 49 [22 – 251] | 146 [40 – 394] | 202 [81 – 395] |
| AST (U/L) | 145 [55 – 621] | 177 [52 – 545] | 136 [51 – 264] |
| Total bilirubin (µmol/L) | 1·8 [0·8 – 3·7] | 1·2 [0·6 – 2·3] | 1·5 [0·6 – 2·8] |
| ALP (U/L) | 126 [74 – 205] | 128 [80 – 223] | 144 [85 – 253] |
| INR | 1·58 [1·30 – 2·07] | 1·47 [1·25 – 1·96] | 1·35 [1·20 – 1·63] |

SALI: Sepsis-associated liver injury; MIMIC-IV: Medical Information Mart for Intensive Care-IV database; SD: Standard deviation; IQR: Interquartile range; SOFA: Sequential Organ Failure Assessment score; ICU: Intensive care unit; ALT: Alanine aminotransferase; AST: Aspartate aminotransferase; ALP: Alkaline phosphatase; INR: International normalized ratio

**Supplementary Table 16: Analyses with additional adjustment for suspected source of infection: Stratification of SALI patients in the MIMIC-IV cohort using Cox regression analysis**

| | | MIMIC-IV Cohort | |
|---|---|---|---|
| | | **Harzard Ratio (95% CI)** | **p-value** |
| **SALI** | **Diagnosis of SALI** | 1·83 (1·66-2·02) | <0·0001 |
| **De Ritis ratio** | **De Ritis ≥2** | 2·46 (2·18-2·77) | <0·0001 |
| | **2 > De Ritis >1** | 1·56 (1·37-1·78) | <0·0001 |
| | **De Ritis ≤1** | 0·86 (0·68-1·09) | 0·21 |
| **R-factor** | **R ≥ 5** | 2·19 (1·93-2·49) | <0·0001 |
| | **5 > R ≥2** | 1·43 (1·12-1·82) | 0·0054 |
| | **R < 2** | 1·53(1·36-1·72) | <0·0001 |
| **ALT Concentration** | **ALT ≥ 5 ULN** | 2·16 (1·91-2·44) | <0·0001 |
| | **5 ULN > ALT ≥ 2 ULN** | 1·42 (1·16-1·73) | 0·0011 |
| | **ALT < 2 ULN** | 1·41 (1·23-1·60) | <0·0001 |

SALI: Sepsis-associated liver injury; MIMIC-IV: Medical Information Mart for Intensive Care-IV database; CI: Confidence interval; ALT: Alanine aminotransferase; ULN: Upper limit of normal

**Supplementary Table 17:** **Stratification of SALI Patients with medical or surgical admission in the MIMIC-IV cohort using Cox regression analysis**

| | | Medical admission | | Surgical admission | |
|---|---|---|---|---|---|
| | | Harzard Ratio (95% CI) | p-value | Harzard Ratio (95% CI) | p-value |
| **De Ritis ratio** | De Ritis ≥2 | 2·52 (2·21-2·87) | <0·0001 | 2·19 (1·60-3·00) | <0·0001 |
| | 2 > De Ritis >1 | 1·63 (1·42-1·87) | <0·0001 | 1·07 (0·72-1·59) | 0·74 |
| | De Ritis ≤1 | 0·82 (0·64-1·06) | 0·13 | 0·86 (0·41-1·83) | 0·71 |

SALI: Sepsis-associated liver injury; MIMIC-IV: Medical Information Mart for Intensive Care-IV database; CI: Confidence interval
